# *Limosilactobacillus reuteri* alleviates proinflammatory T-cell-mediated liver injury and transcriptomic changes in immunocompromised mice

**DOI:** 10.3389/fimmu.2026.1713120

**Published:** 2026-03-03

**Authors:** Ana Fadhel Alvarez, Zheng Yin, Beanna Okeugo, Alexander Banerjee, Meng Luo, Christopher M. Taylor, Salomea Giorgberidze, Vaishali Harne, Rambabu Majji, Melissa N. Munroe, Ji Ho Suh, Stephen T. C. Wong, Kang Ho Kim, Hari Krishna Yalamanchili, Suhair Al Salihi, Jon Marc Rhoads, Yuying Liu

**Affiliations:** 1 Division of Gastroenterology, Department of Pediatrics, McGovern Medical School at UTHealth Houston, Houston, TX, United States; 2 Systems Medicine and Bioengineering Department, Houston Methodist Neal Cancer Center, Houston, TX, United States; 3 Department of Pathology and Laboratory Medicine, McGovern Medical School at UTHealth Houston, Houston, TX, United States; 4 Microbial Genomics Resource Group, Department of Microbiology, Immunology and Parasitology, Louisiana State University Health Sciences Center, New Orleans, LA, United States; 5 Jan and Dan Duncan Neurological Research Institute, Texas Children's Hospital, Houston, TX, United States; 6 Department of Pediatrics, USDA/ARS Children's Nutrition Research Center, Baylor College of Medicine, Houston, TX, United States; 7 Department of Anesthesiology, Critical Care and Pain Medicine, McGovern Medical School at UTHealth Houston, Houston, TX, United States

**Keywords:** autoimmunity, gut microbiota, hepatic transcriptome, inflammation, probiotic-modulated T cell, probiotics, RAG1 knockout mice, Treg deficiency

## Abstract

**Background:**

A deficiency of immunosuppressive regulatory T cells, as seen in scurfy (SF) mice or in IPEX syndrome in humans, can lead to multiorgan inflammation. Oral administration of the probiotic *Limosilactobacillus reuteri* Deutsche Sammlung von Mikroorganismen und Zellkulturen GmbH (DSM) 17938 prolongs survival and reduces Th1- and Th2-associated inflammation in SF mice. It remains unclear how DSM 17938-educated SF-CD4^+^ T cells modulate T-cell–liver communication.

**Methods:**

To characterize CD4^+^ T cells from SF mice orally administered DSM 17938 (Prob-SF-CD4^+^ T cells) and to compare them with CD4^+^ T cells from SF mice (SF-CD4^+^ T cells), cells isolated from SF spleens were adoptively transferred by intraperitoneal (IP) injection into lymphocyte-deficient Recombination-Activating Gene (RAG)-1-deficient (RAG1KO) mice. Liver histological inflammation and macrophages (MΦs), liver sample transcriptomes by RNAseq, and stool microbiota by 16S rRNA sequencing were then assessed in RAG1KO mice.

**Results:**

Prob-SF-CD4^+^ T cells reduced the incidence and severity of liver inflammation and F4/80^+^MΦ infiltration by SF-CD4^+^ T-cell transfer. SF-CD4^+^ T cells upregulated genes and altered RNA splicing factors and events involved in inflammatory pathways, including Toll-like receptor (TLR) cascades, inflammatory cytokines, and death receptor signals. SF-CD4^+^ T cells downregulated genes linked to metabolism, including mitochondrial function, the TCA cycle, lipids, and liver detoxification. Prob-CD4^+^ T-cell transfer reversed SF-CD4^+^ T-cell-induced gene changes in inflammatory and metabolic interactive clusters while modulating distinct genes involved in TLR regulation and the cell cycle. CD4^+^ T-cell transfer altered gut microbial diversity compared with RAG1KO mice without CD4^+^ T-cell transfer. Prob-SF-CD4^+^ T-cell transfer exclusively increased the relative abundance (RA) of *Incertae_sedis* and reduced the RA of *Clostridia_vadinBB60_group* in the stool of RAG1KO mice.

**Conclusions:**

Inflammatory lymphocytes (CD4^+^ T cells) can perpetuate an exaggerated immune response in an immunologically naïve host. Feeding with DSM 17938 modulated T cells and allowed them to provide beneficial effects to the recipient. We observed activation of multiple genes and their interactions. These findings suggest that probiotics or probiotic-modulated T cells could be further explored as therapeutic options for autoimmune liver diseases.

## 1 Introduction

The immune system is composed of innate and adaptive components that protect the body from foreign pathogens. The homeostasis of the adaptive immune system depends on regulatory T cells (Tregs), a subtype of CD4^+^ cells with immunosuppressive properties (1). Treg functions are mediated by the expression of the transcription factor Foxp3^+^ and the interleukin (IL)-2 receptor α-chain (CD25), preventing the development of autoimmune and autoinflammatory conditions (1). Treg deficiency caused by *Foxp3* gene mutation or depletion activates inflammatory and autoreactive cells, leading to a cascade of events resulting in cytokine production and widespread immune dysregulation (2–5), exemplified by the scurfy (SF) mouse, which exhibits multiorgan inflammation and early death. The SF mouse mimics a human disease with immune dysregulation, polyendocrinopathy, enteropathy, X-linked inheritance (called the IPEX syndrome) (6).

Autoimmune liver diseases (AILD) include autoimmune hepatitis (AIH), primary sclerosing cholangitis (PSC), and primary biliary cholangitis (PBC). Liver damage in SF mice has been compared to AILD in humans based on histological patterns and increases in autoantibodies (7). Autoimmune liver attack involves autoreactive T-cell differentiation, B-cell maturation, and macrophage activation, driven by cytokines and autoantibodies. Antigens presented by Antigen-Presenting Cells (APCs) (such as B cells, dendritic cells [DCs]) to naive CD4^+^ T cells in lymph nodes or the liver activate them into Th1 cells, which release interferon (IFN)-γ and IL-2 and activate CD8^+^ T cells and macrophages (MΦ), or into Th2/Th17 cells, which produce IL-4, IL-13, IL-21, and IL-17 to help B cells generate autoantibodies and trigger inflammation, leading to liver inflammation and damage (8). Additionally, innate-like T cells—including natural killer T cells (NKT), γδ T cells, and mucosal-associated invariant T (MAIT) cells—are highly abundant in the liver (9) and can effectively process incoming antigens and release large amounts of cytokines (10).

Current AIH treatment primarily focuses on immunosuppression with corticosteroids and other immunosuppressants (11, 12). In some patients, liver damage progresses all the way to liver failure, ultimately requiring liver transplantation. Notably, even after transplantation, recurrent portal inflammation often develops, which is associated with T-cell activation.

Probiotics educate T cells in the gut by promoting Tregs and suppressing inflammatory T cells such as Th1/Th2/Th17 cells, creating immune tolerance and balance through the activation of DCs, generation of T-cell-modulating metabolites (e.g., short-chain fatty acids [SCFAs], adenosine/inosine), and/or direct microbial signals that influence T-cell differentiation. These effects translate systemically to reduce autoimmune issues, promote anti-inflammatory responses, and regulate overall immune homeostasis, impacting distant sites such as the liver and brain (13). Foxp3^+^ deficiency results in Th1- and Th2-driven autoimmunity, and an altered gut microbial community has also been shown to contribute to immune dysregulation (3).

Oral administration of probiotic *Limosilactobacillus reuteri* DSM 17938 (DSM 17938) remodels the microbiome and ameliorates Th1-/Th2-associated inflammation in SF mice (3). We have shown that adenosine/inosine from this probiotic interacts with the adenosine receptor 2A (A_2a_) on inflamed T cells to reduce inflammation in SF mice (3, 14). Furthermore, DSM 17938-educated Tregs have been shown to exhibit more effective anti-inflammatory properties than naïve Tregs in the intestine of neonatally stressed mice (15). DSM 17938 also improved Foxp3-deficiency-induced liver inflammation and dyslipidemia in mice (16).

RAG-1-deficient (RAG1KO) mice lack mature B and T lymphocytes due to arrest of cell differentiation at an early stage (17). This mouse model provides a tool for investigating probiotic-modulated CD4^+^ T cells and their effects on liver MΦ and the pathobiological mechanisms of probiotic-T-cell function. Therefore, in this study, CD4^+^ T cells from SF mice, with or without probiotic exposure, were isolated and transferred into RAG1KO mice to analyze their effects on T-cell-mediated hepatitis and liver transcriptomes.

## 2 Materials and methods

### 2.1 Mice

Wild-type (WT) C57BL/6J (000664), heterozygous B6.Cg-Foxp3sf/J female (004088), and RAG1KO homozygous B6.129S7-Rag1^tm1Mom^/J (002216) mice, 6–8 weeks old, were purchased from the Jackson Laboratory (Bar Harbor, ME, USA) and allowed to acclimatize for 2 weeks before breeding pairs were established to generate WT, RAG1KO, and SF mice (B6.Cg-Foxp3sf/Y). The mice were housed under a 12-h light/12-h dark cycle at temperatures of 18°C–23°C with 40%–60% humidity. Food and water were provided *ad libitum* in a specific pathogen-free (SPF) animal facility at the University of Texas Health Science Center at Houston (UTHealth Houston). SF mice were generated by breeding a WT male with a heterozygous B.Cg-Foxp3sf/J female mouse. As the *Foxp3* gene is located on the X chromosome, SF features were observed only in males, including scaly skin on the ears, eyes, and tails; deformed ears beginning on day of life 13; and early mortality around days 24–28 of life (3). SF mice were collected from at least three different cages per experimental group. This study was conducted in accordance with the recommendations of the Guide for the Care and Use of Laboratory Animals of the National Institutes of Health (NIH). The Institutional Animal Care and Use Committee (IACUC) of UTHealth approved the study (protocol number: AWC-022-0112).

### 2.2 Probiotic preparation

Probiotic DSM 17938, provided by BioGaia AB (Stockholm, Sweden), was anaerobically cultured in deMan–Rogosa–Sharpe (MRS) medium (Difco™ Lactobacilli MRS Broth, BD, Franklin Lakes, NJ, USA) at 37°C for 24h. It was then plated on MRS agar (Difco™ Lactobacilli MRS Agar) in serial dilutions and grown at 37°C for 48–72 h. Quantitative analysis of bacteria in the culture medium was performed by comparing the optical density (OD) at 600 nm of cultures at known concentrations using a standard curve generated from bacterial colony-forming units (CFU)/mL grown on MRS agar. Cultured DSM 17938 bacteria were suspended in sterile saline at the calculated CFU required for each feeding and prepared daily.

### 2.3 Adoptive transfer of CD4^+^ T cells from SF mice with or without probiotic treatment to RAG1KO

SF mice were fed DSM 17938 (10^7^ CFU/day in 100 µL [SFL]) or sterile saline (100 µL [SFC]) via intragastric administration daily from day 8 of life (d8) to d20. On day 21, mice were euthanized with 5% inhaled isoflurane, followed by cervical dislocation to ensure death prior to vital organ harvest. CD4^+^ T cells were isolated from splenocytes using the EasyStem™ Mouse CD4^+^ T Cell Isolation Kit (STEMCELL Technologies, Vancouver, Canada), following the manufacturer’s protocol. CD4^+^ T-cell purity was subsequently assessed by flow cytometry. Non-CD4^+^ T cells (CD4^−^ T cells) and naïve-like non-CD4^+^ T cells (CD4^−^CD45RB^high^) were removed from splenocytes of SF mice. The purity of CD4^+^ T cells used for adoptive transfer was > 90% (**Supplementary Figure S1**). Purified CD4^+^ T cells were centrifuged at low speed (300×*g*) for 10 min, after which the supernatant (containing cell debris or dead cells, if present) was carefully removed. The cell pellets were gently suspended in sterile saline, and the cells were stained with 0.4% of trypan blue to count blue-stained (dead) and unstained (live) cells using a hemocytometer under a light microscope at low magnification. After determining the number of viable cells, the cells were immediately transferred to RAG1KO mice (10-week-old, both male and female) via intraperitoneal (IP) injection at a dose of 1 × 10^6^ live cells/mouse. CD4^+^ T cells isolated from the spleens of SF mice fed probiotic DSM 17938 were designated as probiotic-modulated SF-CD4^+^ T cells (Prob-SF-CD4^+^ T cells), whereas CD4^+^ T cells isolated from the spleens of SF mice fed saline were designated as SF-CD4^+^ T cells for comparison.

The RAG1KO mice that received Prob-SF-CD4^+^ T cells were designated RAG1KOATSFL, whereas RAG1KO mice that received SF-CD4^+^ T cells were designated RAG1KOATSFC. After 4 weeks of adoptive transfer (AT), RAG1KO mice were euthanized with an overdose (5%) of inhaled isoflurane, and liver tissues were collected. Part of the liver from each mouse was fixed for histological evaluation; another section was freshly frozen and immediately stored at – 80 °C for RNA sequencing (RNAseq) analysis. RAG1KO mouse stools were collected and immediately stored at − 80 °C for microbiota analysis.

The number of mice used for each analysis in each study group is indicated in the figure legends and **Supplementary Table S1**.

### 2.4 Flow cytometry analysis of CD4^+^ T-cell activation markers

**Figure 1:**
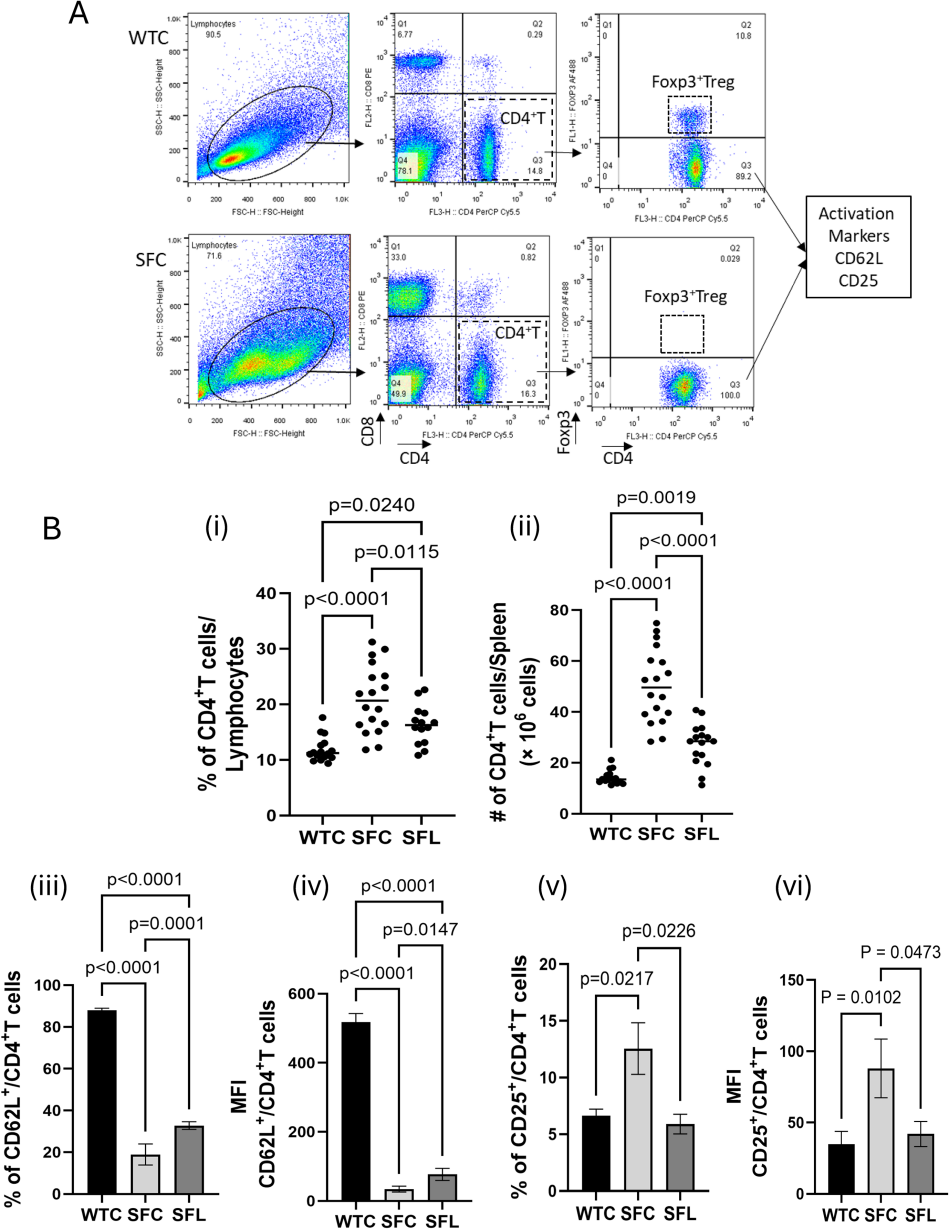
CD4^+^ T-cell analysis of splenocytes from SF mice. **(A)** Gating strategy for the identification of lymphocyte and cell subtype populations, including CD4^+^ T cells, CD8^+^ T cells, Foxp3^+^, Foxp3^−^, and, among Foxp3^−^CD4^+^ T cells, cells expressing activation markers, including CD62L and CD25. **(B)** CD4^+^ T-cells and CD4^+^ T-cell subsets compared across different mouse groups: (i) % of CD4^+^ T cells/lymphocytes; (ii) absolute number (× 10^6^) of CD4^+^ T cells/spleen; (iii) % of CD62L^+^ among Foxp3^−^CD4^+^ T cells; (iv) MFI of CD62L^+^ among Foxp3^−^CD4^+^ T cells; (v) % of CD25^+^ among Foxp3^−^CD4^+^ T cells; (vi) MFI of CD25^+^ among Foxp3^−^CD4^+^ T cells. Each dot represents a mouse (*n* = 13–16 mice per group). Statistically significant *p*-values (*p* < 0.05) are indicated in the figures.

Single-cell suspensions from the spleen of SF mice, with or without probiotic treatment, were stained with fluorescence-labeled antimouse antibodies for surface staining, including CD4 (GK1.5) conjugated with peridinin chlorophyll protein/cyanine 5.5 (PerCP/Cy5.5), CD8a (53-6.7) conjugated with phycoerythrin (PE), CD25 (PC61) conjugated with Alexa Fluor 700 (AF700), and CD62L (MEL-14) conjugated with Pacific Blue (PB); Foxp3 (FJK-16s) conjugated with Alexa Fluor 488 (AF488) was used for intracellular staining using the Foxp3/Transcription Factor Staining Buffer Set (eBioscience, San Diego, CA, USA) to permeabilize cells according to the manufacturer’s instructions. All antibodies were purchased from BioLegend (San Diego, CA, USA). Data from all samples were acquired on a Gallios Flow Cytometer (Beckman-Coulter Life Sciences, Indianapolis, IN, USA) and analyzed using FlowJo software version 10.8.1 (BD). We first identified CD4^+^ T cells among lymphocyte populations and confirmed Foxp3^+^ Tregs and Foxp3^−^ non-Tregs. Among Foxp3^−^ non-Tregs, we further identified T-cell activation using markers including CD62L and CD25 (**Figure 1A**). The percentage of CD62L- and CD25-positive cells within the defined cell population was calculated. Mean fluorescence intensity (MFI), which quantifies the average brightness or expression level of a fluorescent signal, was measured for both CD62L- and CD25-positive cell populations using the geometric mean.

### 2.5 Liver tissue histological evaluation and immunohistochemistry of MΦ

Liver tissues were fixed in formalin, processed, and stained with hematoxylin and eosin (H&E) by the Histology Laboratory of the Department of Pathology and Laboratory Medicine at McGovern Medical School at UTHealth Houston. Hepatitis evaluation was performed in a blinded manner by two pathologists from the same department. Liver inflammation was graded using the Batts–Ludwig scoring system, with severity ranging from grade 0 (normal) to grade 4 (severe inflammation with bridging necrosis) (18).

For immunohistochemistry (IHC) analysis of MΦ, proteinase K (Abcam AB64220, Waltham, MA, USA) was used for enzymatic antigen retrieval, followed by blocking of endogenous peroxidase using 3% H_2_O_2_ (Sigma-Aldrich, St. Louis, MO, USA) and nonspecific binding with 2.5% goat serum (Vector Laboratories, Newark, CA, USA). A rat monoclonal antimouse antibody F4/80 (MCA497R, Bio-Rad Laboratories, Hercules, CA, USA) was used for staining at a 1:200 dilution and incubated at 4°C overnight. Secondary antibody treatment was performed using the ImmPRESS HRP Goat Anti-Rat IgG Polymer Detection Kit (MP-7444, Vector Laboratories) for 30 min at room temperature, followed by the addition of DAB substrate (SK-4105, Vector Laboratories) and counterstaining with hematoxylin. F4/80-positive cells in liver sections were quantified by counting the number of positive cells per high-power field (HPF) at × 400 magnification across 10 randomly selected fields using light microscopy.

### 2.6 RNA sequencing for the liver tissues from RAG1KO mice

Total RNA was extracted from mouse liver tissues using the RNAeasy Mini Kit (QIAGEN, Germantown, MD, USA) according to the manufacturer’s instructions. RNAseq was performed at the Cancer Genomics Center (CGC) at UTHealth (CPRIT RP240610). The total RNA was quality-checked using the Agilent RNA 6000 Pico kit (5067–1513) on an Agilent Bioanalyzer 2100 (Agilent Technologies, Santa Clara, CA, USA). Libraries were prepared with NEBNext Ploy(A) mRNA Magnetic Isolation Module (E7490L, New England Biolabs, Ipswich, MA, USA), the NEBNext Ultra II Directional RNA Library Prep Kit for Illumina (E7760L, New England Biolabs), and the NEBNext Multiplex Oligos for Illumina (E6609S, New England Biolabs) following the manufacturer’s instructions. The quality of the final libraries was examined using the Agilent High-Sensitivity DNA Kit (5067–4626) on an Agilent Bioanalyzer 2100, and library concentrations were determined by quantitative PCR (qPCR) using Collibri Library Quantification Kit (A38524500, Thermo Fisher Scientific, Waltham, MA, USA). The libraries were pooled evenly and subjected to paired-end 150-cycle sequencing on an Illumina NovaSeq System (Illumina Inc., San Diego, CA, USA). Liver RNA samples of high quality (RNA Integrity Number > 7) were used for RNA sequencing.

### 2.7 Transcriptome analysis of the liver of RAG1KO mice

#### 2.7.1 RNA sequencing data processing

Raw sequencing reads (RAG1KOnoAT, *n* = 4; RAG1KOATSFC, *n* = 6; and RAG1KOATSFL, *n* = 6) underwent comprehensive quality assessment using FastQC v0.11.9 (19), which evaluated read quality metrics and adapter content. Subsequently, read preprocessing and adapter removal were performed using fastp v0.20.0 (20). High-quality processed reads were aligned to the mouse reference genome Genome Reference Consortium Mouse Build 38 (GRCm38), release v23, using STAR v2.7.10a (21). The Spliced Transcripts Alignment to a Reference (STAR) alignment index was constructed using FASTA sequences and GTF annotation files obtained from the Gene Encyclopedia Of DNA Elements (GENCODE) portal. Three biological replicates (*n* = 3) per group were included, displaying uniformly high per-base sequence quality with Phred scores exceeding 33 (**Supplementary Figure S2**) and total mapping rates > 68% (**Supplementary Table S2**), which were subsequently used for analyses of differentially expressed genes (DEGs), pathways, and RNA diversity. In addition, samples separated clearly by genotype in PCA using normalized gene counts (**Supplementary Figure S3**), reflecting distinct transcriptomic signatures consistently captured across biological replicates.

#### 2.7.2 Identification of DEGs

Raw gene-level read counts were quantified using STAR (21) alignment with the quantMode GeneCounts parameter. Differential gene expression analysis was performed with DESeq2 (22) following count data normalization. To ensure robust statistical analysis, genes with mean read counts below 50 across all samples were excluded from downstream analysis. Significantly DEGs were identified using stringent criteria: an adjusted *p*-value ≤ 0.05 combined with a log_2_ fold change threshold of ± 0.263, corresponding to a minimum 20% expression change (23). Genes were prioritized for validation using a relatively low log_2_fold change (log_2_FC) cutoff (e.g., ~ 0.2, corresponding to about a 1.15-fold change) in combination with a significant adjusted *p*-value, allowing identification of a broad set of potential genes for future validation, as subtle expression differences may still be biologically meaningful.

#### 2.7.3 Analysis of alternative splicing events

Alternative splicing patterns were comprehensively analyzed using replicate Multivariate Analysis of Transcript Splicing (rMATS) v4.1.2 (24) with STAR-generated BAM alignment files and GENCODE v23 gene annotations for the mouse reference genome GRCm38. The analysis included five distinct alternative splicing event categories: exon skipping (SE), intron retention (RI), mutually exclusive exons (MXE), alternative 5′ splice sites (A5SS), and alternative 3′ splice sites (A3SS). Statistical significance was determined using a FDR threshold of ≤ 0.05, while biological relevance was assessed based on inclusion-level differences (ILD) of ≥ 0.2 or ≤ − 0.2, representing substantial changes in exon inclusion vs. exclusion patterns.

#### 2.7.4 Pathway analysis and visualization

ConsensusPathDB (release MM11 for mouse) was used to identify pathways overrepresented among DEGs and differentially spliced genes (DSGs). To gain insights from the pool of enriched pathways from various data sources, the Broad Institute Morpheus platform was used to produce pathway-membership heatmaps for top-ranked overexpressed pathways with related biological functions. Hierarchical clustering of the pathway-membership heatmaps identified key groups of genes driving the enrichment of critical pathways. STRING (v12.0) database was applied to create protein–protein interaction networks among those key gene groups. Customized scripts using the enhancedGraphics app within Cytoscape were employed to incorporate our DEG/DSG data with the protein–protein interaction (PPI) networks and visualize the resulting union networks (25, 26).

#### 2.7.5 Verification of gene expressions in the liver of RAG1KO mice with or without adoptive transfer of SF-CD4^+^ T cells using a quantitative real-time polymerase chain reaction

The total RNA (200 ng) was reverse transcribed using iScript Reverse Transcription Supermix for RT-qPCR (Bio-Rad Laboratories, Hercules, CA, USA). Quantitative real-time polymerase chain reaction (qRT-PCR) was performed using iTaq Universal SYBR Green Supermix (Bio-Rad Laboratories, Hercules, CA, USA) on the CFX Opus 384 Real-Time PCR System (Bio-Rad Laboratories, Hercules, CA, USA). Glyceraldehyde-3-phosphate dehydrogenase (GAPDH) was used as a housekeeping gene to normalize gene expression. The genes evaluated included Lipocalin-2 (Lcn2), insulin-like growth factor-binding protein 1 (Igfbp1), Uridine Diphosphate (UDP) glucuronosyltransferase 2 family, polypeptide B38 (Ugt2b38), and Orosomucoid 2 (Orm2). All qPCR primers are listed in **Supplementary Table S3**.

### 2.8 Stool microbial community analysis

Stool samples from mice, including WT (*n* = 9), RAG1KO (*n* = 9), RAG1KOATSFC (*n* = 7), and RAG1KOATSFL (*n* = 7), were used for microbiota analysis. Stool DNA was extracted using the QIAamp Fast DNA Stool Mini Kit (QIAGEN, Germantown, MD, USA) according to the manufacturer’s protocol. Sequencing and bioinformatics analyses were performed at the Microbial Genomics Resource Group at Louisiana State University Health Sciences Center. The 16S ribosomal DNA hypervariable region V4 was PCR-amplified using primers V4F GTGCCAGCMGCCGCGGTAA and V4R GGACTACHVGGGTWTCTAAT with Illumina adaptors and molecular barcodes to produce amplicons. Samples were sequenced on an Illumina MiSeq (Illumina Inc.) using a 500-cycle V2 sequencing kit to produce 2 × 250 paired-end reads. The forward and reverse-read files were processed using DADA2 (27) and pipelined in QIIME2 (28). Amplicon sequence variants were taxonomically classified using the SILVA v138 database (29). Bacterial alpha and beta diversity metrics, as well as taxonomic community assessments, were performed using QIIME2.

### 2.9 Statistical analysis

Adjusted *p*-values were used for gene analysis with the specified analytic techniques described above. For analyzing the percentage and MFI of immune cells, the number of MΦ counts, and the relative abundance of stool bacteria, significance was determined using one-way ANOVA for multiple comparisons with Tukey’s *post-hoc* tests. Data are represented as means ± SD. *p*-values of < 0.05 were considered statistically significant. Analyses were performed using GraphPad Prism version 9.4.1 (GraphPad Software, San Diego, CA, USA).

## 3 Results

### 3.1 Adoptive transfer of Prob-SF-CD4^+^ T cells ameliorated liver inflammation in immunocompromised RAG1KO mice

**Figure 2:**
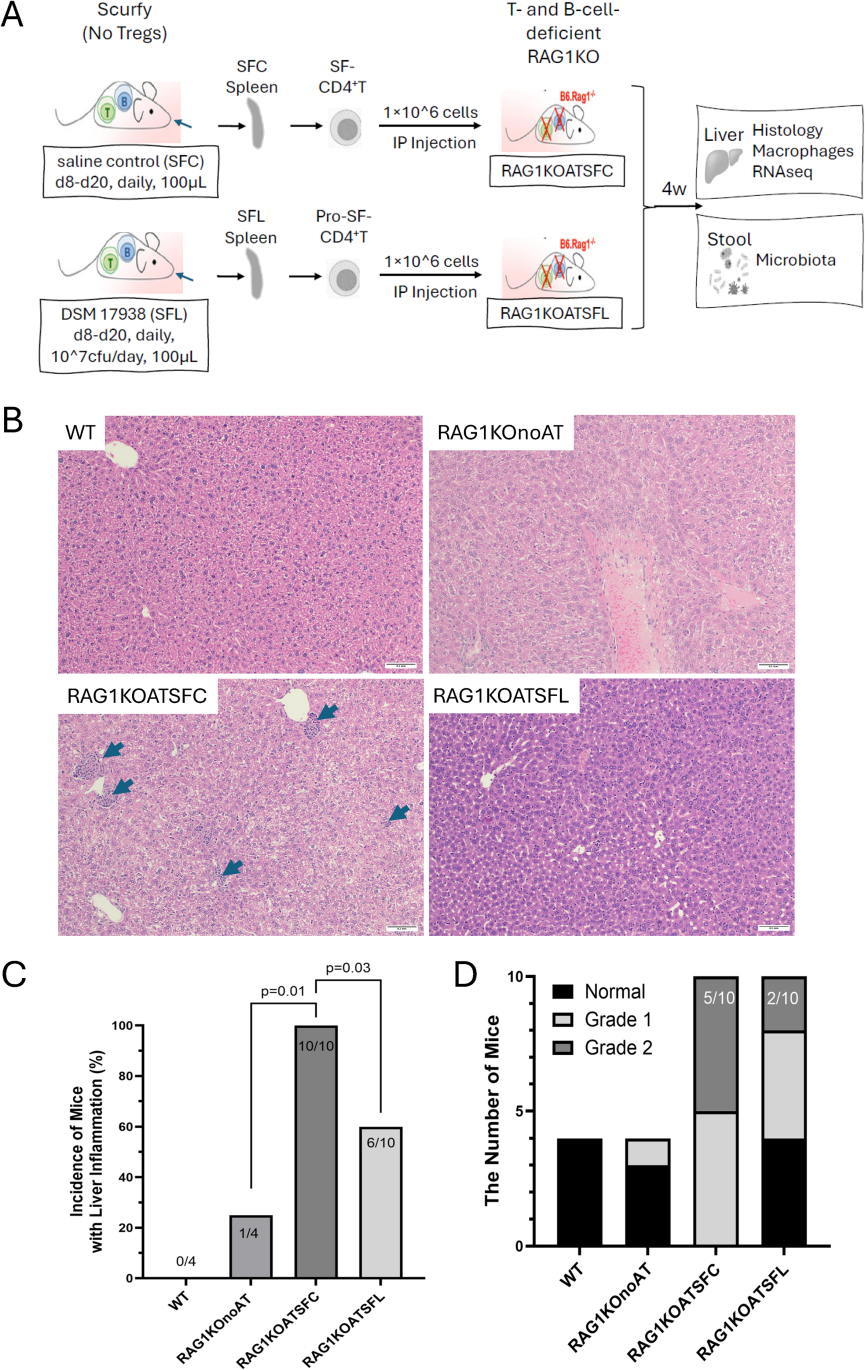
Liver histological evaluation of RAG1KO mice after adoptive transfer of SF-CD4^+^ T cells isolated from SF mice fed saline control (SFC) (the group was designated as RAG1KOATSFC) or Prob-SF-CD4^+^ T cells isolated from SF mice fed *L. reuteri* DSM 17938 (SFL) (the group was designated as RAG1KOATSFL). **(A)** Experimental scheme. **(B)** Representative liver sections of WT, RAG1KOnoAT, RAG1KOATSFC, and RAG1KOATSFL mice with H&E staining. Arrows indicate inflammatory cell infiltration. Scale bar = 0.1 mm (100 µm) with × 100 magnification. **(C)** Incidence of mice with liver inflammation (%), comparing the different groups. **(D)** Severity of liver inflammation was evaluated using the Batts–Ludwig scoring system, with severity ranging from grade 0 (normal) to grade 4 (severe inflammation with bridging necrosis), comparing the different groups, *n* = 4–10 mice per group.

SF mice have increased circulating and tissue-resident inflammatory T cells, resulting in multiorgan inflammation (3, 30). As we planned to use CD4^+^ T cells isolated from the spleens of SF mice for an adoptive transfer study, we first analyzed CD4^+^ T cells using markers to assess their proliferative and activated status in splenocytes from SF mice treated with the probiotic DSM 17938. CD62L (l-selectin), a cell surface adhesion molecule, is downregulated by shedding from the cell membrane once a T cell is activated (31). CD25, a component of the IL-2 receptor, is present on the surface of immune cells, with high expression on activated lymphocytes (32). Here, we showed that both the proportion (**Figure 1B**i) and absolute numbers (**Figure 1B**ii) of CD4^+^ T cells in the spleen of SF mice were significantly increased. This increase could be reversed by oral administration of DSM 17938 (**Figures 1B**i, ii). Quantitatively, SF-CD4^+^ T cells exhibited significantly altered activation biomarkers, as shown by a reduced percentage of CD62L^+^ (**Figure 1B**iii) and reduced MFI of CD62L^+^ T cells (**Figure 1B**iv), alongside an increased percentage of CD25^+^ (**Figure 1B**v) and increased MFI of CD25^+^ T cells (**Figure 1B**vi). The proportion and MFI of activated T cells were also reversed by DSM 17938 (**Figures 1B**iii–vi). We previously demonstrated that DSM 17938 reduced the number of IFN-γ- and IL-4-producing splenocytes (3). These results indicate that oral administration of DSM 17938 can modulate SF-CD4^+^ T cells in the spleen of SF mice. In the current investigation, to further identify the functions of SF-CD4^+^ T cells and probiotic-modulated SF-CD4^+^ T cells (Prob-SF-CD4^+^ T cells), these CD4^+^ T cells were isolated from the spleens of SF mice and immediately injected into RAG1KO mice. RAG1KO mice that received SF-CD4^+^ T cells were designated RAG1KOATSFC, whereas RAG1KO mice that received Prob-SF-CD4^+^ T cells were designated RAG1KOATSFL. Liver histology, MΦs, transcriptomes, and stool microbiota of these RAG1KO mice were analyzed (**Figure 2A**).

We found that RAG1KOATSFC mice demonstrated liver pathology, exhibiting multifocal periportal lymphocytic infiltration with degenerating hepatocytes (**Figure 2B**). The incidence of liver inflammation in RAG1KOATSFC mice was 100% (10/10), compared to 60% (six of 10) in RAG1KOATSFL mice (*p* = 0.025) (**Figure 2C**). We assessed the severity of liver inflammation by scoring histological samples from grade 0 (normal) to grade 4 (severe inflammation with bridging necrosis). The maximum severity observed in this study was Grade 2. In the RAG1KOATSFC group, five mice exhibited grade 1, and five mice exhibited grade 2 histologic severity. In contrast, in mice receiving Prob-SF-CD4^+^ T cells (RAG1KOATSFL), only two mice had grade 2 inflammation, four had grade 1 inflammation, and four were normal (**Figure 2D**). These results indicate that the severity of hepatitis was ameliorated by Prob-SF-CD4^+^ T cells in RAG1KO mice, as shown by the comparison of RAG1KOATSFL to RAG1KOATSFC mice (*p* = 0.011). Overall, these findings suggest that DSM 17938 can reduce the inflammatory activation of SF-CD4^+^ T cells.

### 3.2 Prob-SF-CD4^+^ T cells reduce liver MΦ infiltration enhanced by inflammatory SF-CD4^+^ T cells in RAG1KO mice

**Figure 3:**
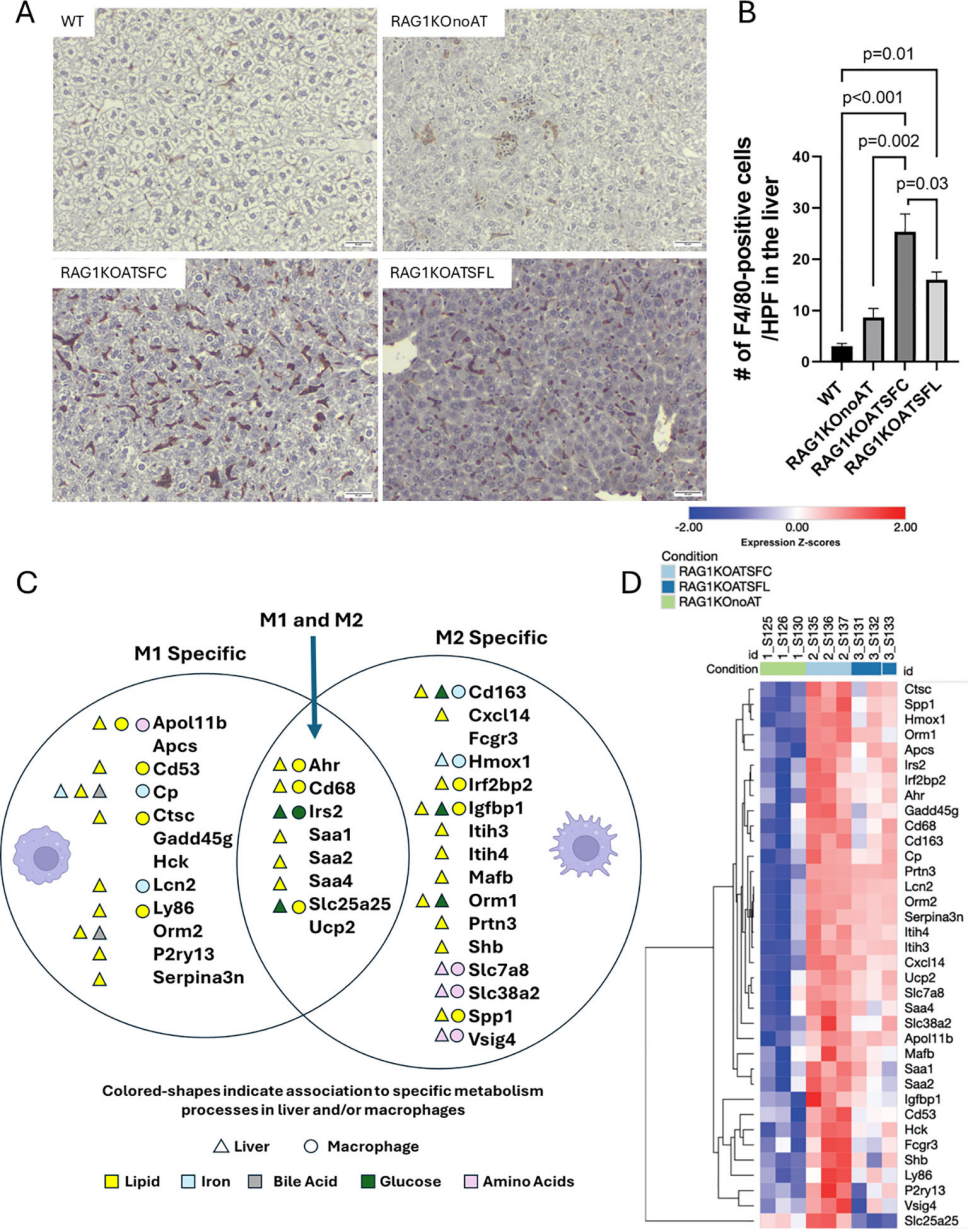
Liver macrophage (MΦ) evaluation from RAG1KO mice after adoptive transfer of CD4^+^ T cells, comparing RAG1KOATSFC or RAG1KOATSFL with WT and RAG1KOnoAT groups. **(A)** Representatives of liver tissue sections from mice stained with F4/80 antibody from WT, RAG1KOnoAT, RAG1KOATSFC, and RAG1KOATSFL groups. F4/80-positive MΦs are indicated by dark brown staining. Scale bar = 50 µm with × 200 magnification. **(B)** Quantitation of the number of F4/80-positive cells per high-power field (HPF) in the liver (counted under × 400 magnification), comparing the different groups (*n* = 4–10 mice per group). **(C)** Liver genes that were upregulated by SF-CD4^+^ T-cell transfer and downregulated by probiotic-modulated SF-CD4^+^ T (Prob-SF-CD4^+^ T)-cell transfer. The diagram shows genes associated with MΦ polarization, categorized by their association with M1, M2, or both phenotypes. Colored shapes indicate gene associations with specific metabolic pathways: lipid (yellow), iron (light blue), bile acid (gray), glucose (green), and amino acid (light pink). Triangles represent genes in liver metabolism, and circles represent genes in MΦ metabolism (*n* = 3 mice per group). **(D)** A heatmap displaying the expression of 36 genes associated with MΦ polarization and metabolism across the three study groups: RAG1KOnoAT, RAG1KOATSFC, and RAG1KOATSFL (*n* = 3 mice per group).

In the liver, CD4^+^ T cells can play a key role in polarizing MΦ, primarily by releasing cytokines that either activate or suppress MΦ activity (33). T-cell- and B-cell-deficient RAG1KO mice have been found to have normal MΦs in the liver (34). After introducing CD4^+^ T cells from SF mice, it was previously unknown as to whether these CD4^+^ T cells, especially probiotic-modified CD4^+^ T cells, could affect liver MΦs in these mice. F4/80 antigen expression, a protein encoded by adhesion G protein-coupled receptor E1 (Adgre1) (35) and widely used as a monocyte and MΦ marker in the liver, was quantified (**Figure 3A**). We observed that the number of hepatic F4/80^+^MΦ cells/HPF was significantly increased in RAG1KOATSFC mice, compared with either RAG1KO without T-cell AT (RAG1KOnoAT) (*p* = 0.002) or normal WT (p < 0.001) (**Figures 3A, B**). However, T cells transferred from probiotic-treated donors partially but significantly reduced the number of F4/80^+^MΦ cells/HPF in RAG1KOATSFL mice (*p* = 0.03). These results indicate that liver MΦ are responsive to both transferred inflammatory CD4^+^ cells and CD4^+^ cells modulated by probiotic treatment.

Liver MΦs have emerged as essential players in maintaining hepatic homeostasis, participating in injury and repair processes in both acute and chronic liver diseases (36). They can be broadly categorized into M1 and M2 phenotypes. M1 cells, which are often proinflammatory, produce reactive oxygen and nitrogen compounds and high levels of cytokines, contributing to inflammation and tissue damage. In contrast, M2 cells have anti-inflammatory and tissue-repair functions, playing a key role in the resolution of inflammation and wound healing (37). Liver transcriptome analysis revealed that 36 genes associated with MΦ polarization in RAG1KO mice were significantly upregulated by AT SF-CD4^+^ T cells compared with RAG1KOnoAT mice; however, these genes were downregulated by AT Prob-SF-CD4^+^ T cells relative to AT SF-CD4^+^ T cells (**Figure 3D**). As expected, the differences in macrophage number observed across conditions are reflected in gene expression, with most genes in SFL showing lower expression than in SFC (**Figure 3D**). Therefore, when comparing gene expression between SFL and SFC, these genes appear downregulated, but this is most likely due to reduced macrophage infiltration in the liver samples rather than reduced activation/inflammation. Interestingly, among these 36 genes, 12 are known to promote M1 polarization, 16 are associated with M2 polarization, and eight are associated with both M1 and M2 differentiation (**Figure 3C**, **Supplementary Table S4**).

Liver MΦ, such as Kupffer cells (KCs), are also involved in controlling iron and cholesterol balance (38). The altered genes are involved in liver nutrient (lipid, iron, bile acid, glucose, and amino acid) metabolism, while at least 18 of these genes have also been reported to participate in MΦ metabolism. Among them, 10 genes (Apol11b, CD53, Ctsc, Ly86, Ahr, CD68, Slc25a25, Igfbp1, Spp1, Irf2bp2) regulate lipid metabolism; four genes (Cp, Lcn2, CD163, Hmox1) are involved in iron metabolism; four genes (Apol11b, Slc7a8, Slc38a2, Vsig4) participate in amino acid metabolism; and one gene (Irs2) regulates glucose metabolism (**Figure 3C**, **Supplementary Table S4**). These results indicate that Prob-SF-CD4^+^ T cells can alter genes that are associated with MΦ polarization, differentiation, and metabolism, thereby influencing MΦ functions and maintaining a healthy immune balance that impacts liver inflammatory processes.

### 3.3 Transferring SF-CD4^+^ T cells to RAG1KO mice changed liver gene profiles in RAG1KO mice

We further identified individual DEGs and gene-associated functional pathways dysregulated by AT of SF-CD4^+^ T-cells. Our aim was to better understand cellular crosstalk during liver disease progression and to elucidate the regulatory mechanisms of probiotic-educated SF-CD4^+^ T cells. High-quality RNA and RNAseq data from the livers of RAG1KO mice were obtained for analysis from the RAG1KOnoAT, RAG1KOATSFC, and RAG1KOATSFL groups. A heatmap of gene regulation and the full DEG lists of log_2_FC with significantly adjusted *p*-values across the groups are shown in **Supplementary Figure S5** and **Supplementary Tables S5**-**7**. Major findings include broad regulatory effects of SF-CD4^+^ T cells on liver inflammation and metabolism.

#### 3.3.1 SF-CD4^+^ T cells upregulated inflammatory pathways

**Figure 4:**
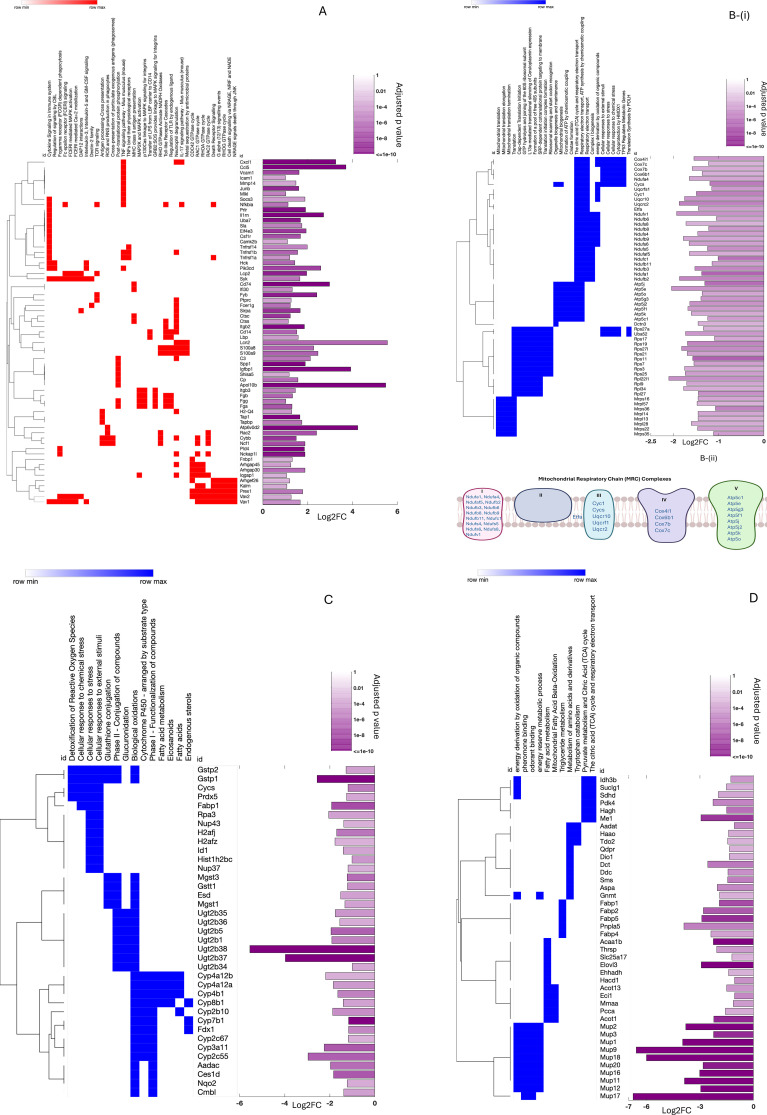
Liver genes altered by SF-CD4^+^ T-cell transfer to RAG1KO mice. Biological pathways were compared between mice receiving T-cell transfer and RAG1KOnoAT controls. **(A)** SF-CD4^+^ T-cell transfer upregulated genes involved in inflammation pathways, with bars demonstrating log_2_FC and significantly adjusted *p*-values. **(B)** SF-CD4^+^ T-cell transfer downregulated genes involved in MRC complexes and mitochondrial formation. **(B)** (i) Mitochondrial pathways with log_2_FC and significantly adjusted *p*-values. **(B)** (ii) Diagram of MRC complexes I, II, III, IV, and V, with downregulated genes indicated for each complex. **(C)** SF-CD4^+^ T-cell transfer downregulated genes involved in the liver detoxification pathway, with log_2_FC and significantly adjusted *p*-values. **(D)** SF-CD4^+^ T-cell transfer downregulated genes that modulate amino acid, lipid, and organic compound metabolism pathways, with log_2_FC and significantly adjusted *p*-values (*n* = 3 mice per group).

Genes upregulated by AT of SF-CD4^+^ T cells were compared with those in mice without AT (RAG1KOATSFC vs. RAG1KOnoAT). Upregulated genes were associated with inflammatory signaling (39), including Toll-like receptor (TLR) cascades; cytokine signaling, including mitogen-activated protein kinase (MAPK) activation, tumor necrosis factor (TNF), IL-3, IL-5, IL-17, and granulocyte-macrophage colony-stimulating factor (GM-CSF); neutrophil degranulation (Lcn2, S100A8, S100A9) (40); GTPase cycles (Rho and Ras superfamily members involved in various cellular processes); and production of reactive oxygen species (ROS) and reactive nitrogen species (RNS) in phagocytes (**Figure 4A**). We also observed activation of receptors associated with phagocytosis, such as Fc gamma receptor (FCGR), antigen presentation, T-cell activation via T-cell receptor (TCR), cell death signaling through neurotrophin-receptor-interacting MAGE-like protein (NRAGE) via the Jun N-terminal kinase (JNK) pathway (41), and C-type lectin receptors (Dectin-2 family) involved in combating fungal infection (**Figure 4A**).

#### 3.3.2 SF-CD4^+^ T cells downregulated mitochondrial formation and mitochondrial respiratory chain complexes

The liver mitochondrial respiratory chain (MRC) complexes (I–V) are crucial for cellular energy production. These complexes work together to transfer electrons from fuel molecules to oxygen, generating a proton gradient that drives ATP synthesis (42). In AIH and other chronic liver diseases, mitochondrial dysfunction—including impaired MRC complex activity—drives inflammation, oxidative stress, and cell death, leading to liver damage (43, 44). We found that AT of SF-CD4^+^ T cells into RAG1KO mice downregulated genes associated with mitochondrial biogenesis, including ribosomal protein-encoded large and small subunits (RPL/RPS), mitochondrial ribosomal protein (MRP), and subunits of the mitochondrial adenosine-triphosphate (ATP) synthase (Atp5) families (**Figure 4B**i). Additional genes downregulated by SF-CD4^+^ T-cell transfer were critical for MRC complexes, affecting complex I (NADH:ubiquinone oxidoreductase [Nduf family]), complex III (ubiquinol-cytochrome *c* reductase [Uqcr10, Uqcrc2, Uqcrcf1] and cytochrome *c* [Cyc1, Cycs]), complex IV (cytochrome *c* oxidase [Cox41l1, Cox6b, 7b/7c]), complex V (mitochondrial ATP synthase [Atp5 family]), and electron transport from II to III (the alpha subunit of the electron transfer flavoprotein [Etfa]) (**Figure 4B**ii).

#### 3.3.3 SF-CD4^+^ T cells downregulated genes participating in liver detoxification

Liver mitochondria not only provide for cellular energy but are also are crucial for liver detoxification by facilitating (i) metabolic processes such as breakdown of fats, carbohydrates, and amino acids, which are essential for detoxification; (ii) efficiency of the tricarboxylic acid (TCA) cycle in removing ammonia, a toxic byproduct of amino acid metabolism; (iii) metabolism of drugs and foreign compounds; and (iv) and glutathione synthesis to neutralize ROS (45). Altered gene expression was observed after AT of SF-CD4^+^ T cells, including (i) glutathione *S*-transferase Pi (Gstp)1/2, peroxiredoxin (Prdx)5, and Cycs, which participate in ROS detoxification; (ii) cytochrome p450 (Cyp) family, arylacetamide deacetylase (Adadc), carboxylesterase 1d (Ces1d), *N*-ribosyldihydronicotinamide:quinone oxidoreductase 2 (Nqo2), and carboxymethylenebutenolidase homolog (Cmbl), involved in biological oxidations; and (iii) the UDP glucuronosyltransferase 2b (Ugt2b) family, such as Ugt2b38 (verified by qRT-PCR; **Supplementary Figure S4**), which participates in glucuronidation responsible for the clearance of many endogenous and exogenous compounds, including drugs and toxins (46). All these genes were suppressed by transferred SF-CD4^+^ T cells in the liver of RAG1KO mice (**Figure 4C**).

#### 3.3.4 SF-CD4^+^ T cells downregulated nutritional metabolite genes

Downregulated genes are associated with the metabolism of amino acids and their derivatives. Specifically, these include genes encoding kynurenine/alpha-aminoadipate aminotransferase (Aadat), 3-hydroxyanthranilate 3,4-dioxygenase (Haao), and tryptophan 2,3-dioxygenase-2 (Tdo2), which play a crucial role in the kynurenine pathway of tryptophan catabolism (47). Other downregulated genes encode a subunit of the mitochondrial enzyme isocitrate dehydrogenase 3b (Idh3b), a subunit of the enzyme succinyl-CoA synthetase (Suclg1), a subunit of the succinate dehydrogenase (Sdhd), pyruvate dehydrogenase kinase 4 (Pdk4), hydroxyacylglutathione hydrolase (Hagh), and malic enzyme 1 (Me1). These genes participate in pyruvate metabolism and the TCA cycle for energy production, particularly within mitochondria (48). Downregulated genes are also associated with lipid metabolism. For example, fatty acid binding protein (Fabp) family regulates fatty acid uptake, storage, and transport; acetyl-CoA acyltransferase 1b (ACAA1b) and enoyl-CoA hydratase/3-hydroxyacyl CoA dehydrogenase (Ehhadh), Acyl-CoA thioesterase 13 (Acot13), and enoyl-CoA delta isomerase 1 (Eci1) are involved in fatty acid oxidation; thyroid hormone responsive (Thrsp) and 3-hydroxyacyl-CoA dehydratases (Hacd) affect lipid synthesis; while propionyl-CoA carboxylase alpha subunit (Pcca) plays a crucial role in the breakdown of certain amino acids and lipids, including cholesterol (49). In addition, SF-CD4^+^ T cells downregulate major urinary protein (Mup) genes, which play significant roles in regulating lipid and glucose metabolism in mice (50) (**Figure 4D**).

### 3.4 Prob-SF-CD4^+^ T cells reversed many SF-CD4^+^ T-cell-induced gene changes in the liver

We further analyzed the expression of genes altered by Prob-SF-CD4^+^ T cells by comparing RAG1KOATSFL mice with RAG1KOATSFC mice. AT of Prob-SF-CD4^+^ T cells to RAG1KO mice mainly affected genes associated with inflammation, cell metabolism, and the cell cycle.

#### 3.4.1 Prob-SF-CD4^+^ T cells downregulated inflammatory genes in the liver of RAG1KO mice

**Figure 5:**
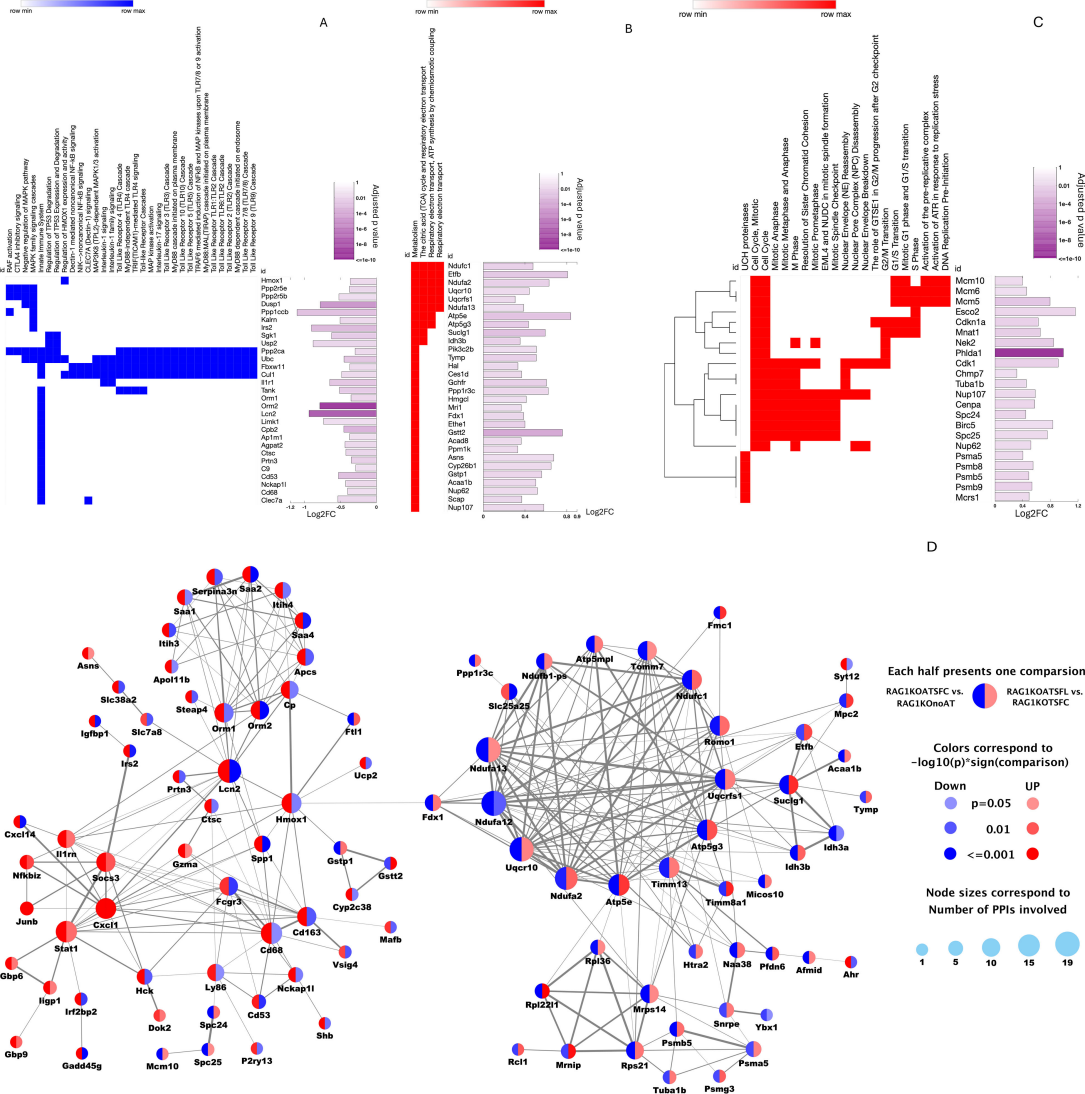
Liver genes altered by Prob-SF-CD4^+^ T-cell transfer to RAG1KO mice compared with SF-CD4^+^ T-cell transfer to RAG1KO mice. **(A)** Prob-SF-CD4^+^ T-cell transfer downregulates genes in inflammation pathways, with bars demonstrating log_2_FC and significantly adjusted *p*-values. **(B)** Prob-SF-CD4^+^ T-cell transfer upregulates genes associated with mitochondrial function and metabolism, with log_2_FC and significantly adjusted *p*-values. **(C)** Prob-SF-CD4^+^ T-cell transfer upregulates genes involved in modulating cell-cycle pathways, with log_2_FC and significantly adjusted *p*-values. **(D)** Gene interaction networks. The cluster on the left shows the inflammatory gene network, whereas the cluster on the right shows the network of metabolism-related genes. Each circle represents one single gene. The left semicircles indicate the effect of SF-CD4^+^ T-cell transfer (RAG1KOATSFC vs. RAG1KOnoAT), and the right semicircles indicate the effect of Prob-SF-CD4^+^ T-cell transfer (RAG1KOATSFL vs. RAG1KOATSFC). Red indicates upregulation, and blue indicates downregulation (*n* = 3 mice per group).

First, we observed that Prob-SF-CD4^+^ T cells downregulated genes associated with inflammatory pathways, including TLR cascades, IL-1 signaling, MAPK activation, and nuclear factor kappa-light-chain enhancer of activated B cell (NF-κB) signaling. The altered genes, however, comprised components of pathways distinct from those upregulated by AT of SF-CD4^+^ T cells (**Figure 5A**). These genes include proinflammatory genes; for example, *Clec7a*, also known as Dectin-1, plays a significant role in detecting fungal pathogens and initiating an immune response (51). The cathepsin C (Ctsc) gene regulates MΦ polarization, neutrophil activation, and cytokine production (52). Ap1m1, a subunit of the adaptor protein complex 1 (AP-1), maintains cellular function (53). Limk1 encodes LIM kinase 1 protein, which influences various cellular processes, particularly those related to actin dynamics, to maintain cell stability (54). The *Lcn2* gene (which encodes the protein Lipocalin-2) stimulates the production of proinflammatory cytokines, thereby amplifying the inflammatory cascade (55). The *Lcn2* gene, upregulated by SF-CD4^+^ T cells and downregulated by Prob-SF-CD4^+^ T cells as shown by RNA-seq, was confirmed by qRT-PCR (**Supplementary Figure S4**).

Notably, five unique genes that regulate NF-κB signaling were downregulated by Prob-SF-CD4^+^ T cells: Cullin 1 (Cul1), the F-box and WD repeat domain-containing 11 gene (Fbxw11), stress-inducible ubiquitin C (Ubc), TRAF family member-associated NF-κB activator (Tank), and a subunit of protein phosphatase 2A (Ppp2ca). Other genes downregulated by Prob-SF-CD4^+^ T-cell transfer exhibit complex roles in regulating inflammation, including NCK-associated protein 1-like (Nckap1l), 1-acylglycerol-3-phosphate *O*-acyltransferase 2 (Agpat2), carboxypeptidase B2 (Cpb2), Ubiquitin-specific peptidase 2 (Usp2), serine/threonine protein kinase 1 (Sgk1), and the protein phosphatase 2A (Ppp2) family (**Figure 5A**).

#### 3.4.2 Prob-SF-CD4^+^ T cells upregulated genes associated with metabolism, including the TCA cycle, ATP synthesis, MRC electron transport, and cell cycle

We discovered that SF-CD4^+^ T cells downregulated over 135 genes associated with liver metabolism (**Figure 4**). In contrast, Prob-SF-CD4^+^ T cells upregulated fewer than 30 genes associated with liver metabolism, primarily in the TCA cycle and MRC electron transport/ATP synthesis (**Figure 5B**). Only five genes overlapped with those downregulated by SF-CD4^+^ T cells (upregulated by Prob-SF-CD4^+^ T cells); these included *Ndufc1*, *Uqcr10*, *Atp5g3*, *Suclg1*, and *Idh3b*. Although Prob-SF-CD4^+^ T cells partially reversed the same metabolic pathways altered by SF-CD4^+^ T cells, the individual genes affected were largely distinct. In addition, Prob-SF-CD4^+^ T cells upregulated genes regulating the cell cycle, including interphase (G1-S-G2) and mitotic (M) phases (**Figure 5C**). The upregulated genes also include ubiquitin carboxyl-terminal hydrolase (UCH) proteinases, which are deubiquitinating enzymes (DUBs) that control the timing and progression of cell division by removing ubiquitin from target proteins (56) (**Figure 5C**).

#### 3.4.3 Genes altered by SF-CD4^+^ T cells that were reversed by Prob-SF-CD4^+^ T cells demonstrated two distinct clusters engaged in complex interactions within each cluster

The interactive gene networks for genes altered by SF-CD4^+^ T cells and reversed by Prob-SF-CD4^+^ T cells were analyzed. These genes formed two distinct clusters. One cluster was related to inflammatory pathways (**Figure 5D**, left cluster), in which most genes were upregulated by SF-CD4^+^ T cells (left semicircles in red), while some were downregulated by Prob-SF-CD4^+^ T cells (right semicircles in blue). Conversely, the other cluster, related to metabolism (**Figure 5D**, right cluster), contained genes that were downregulated by SF-CD4^+^ T cells (left semicircles in blue) and upregulated by Prob-SF-CD4^+^ T cells (right semicircles in red). The genes demonstrated close interactions within each cluster. Notably, these two distinct clusters were connected only by genes encoded by heme oxygenase-1 (Hmox1) from the inflammation cluster and ferredoxin 1 (Fdx1) from the metabolism cluster (**Figure 5D**). *Lcn2*, *Igfbp1*, and *Orm2*, which are upregulated by SF-CD4^+^ T cell transfer but reduced by Prob-SF-CD4^+^ T cell transfer, as shown in the inflammatory network, were verified by qRT-PCR (**Supplementary Figure S4**). This suggests a potential indirect relationship in the context of iron metabolism and inflammation (57).

### 3.5 Adoptive transfer of CD4^+^ T cells from SF mice affected RNA diversity in the liver of RAG1KO mice

**Figure 6:**
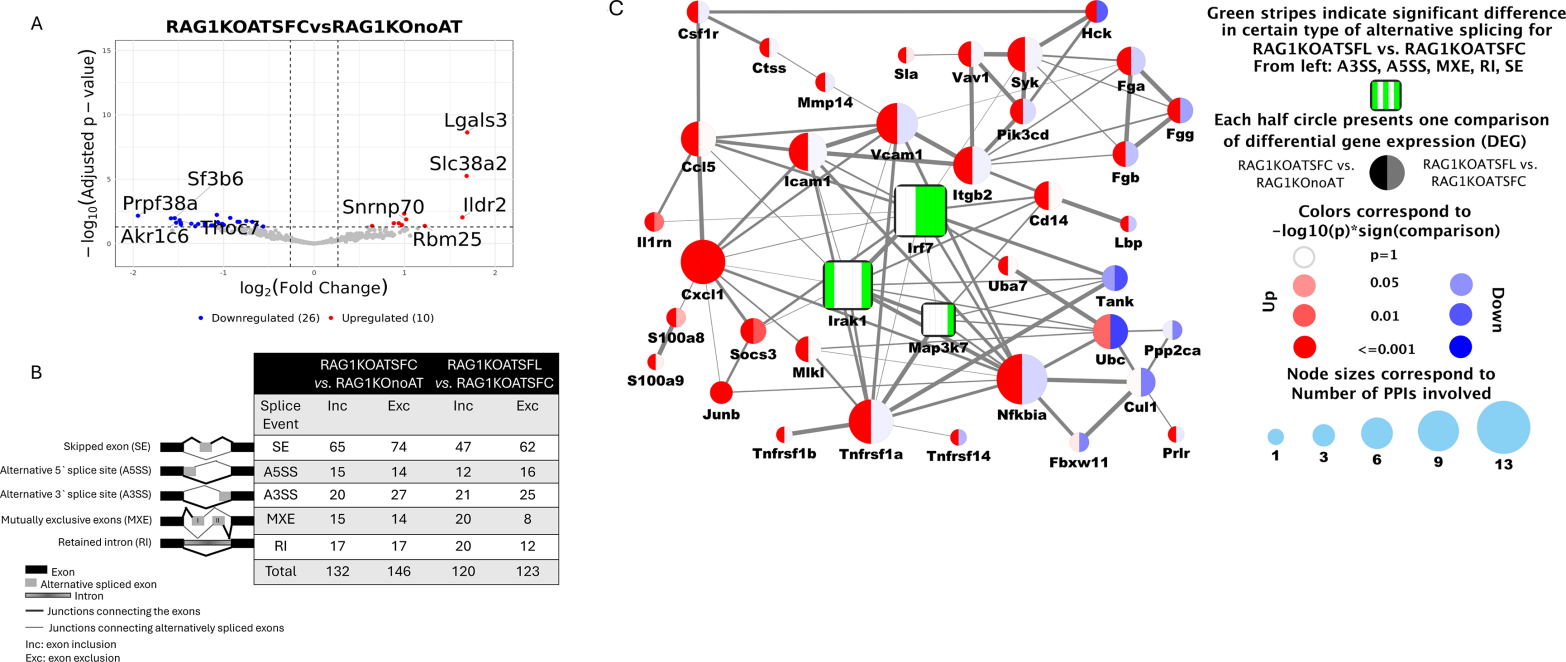
RNA alternative splicing events in DEGs. **(A)** The volcano plot displays DEGs that are also splice factors (SFs). We identified 26 SFs that were downregulated and 10 SFs that were upregulated in the comparison between RAG1KOATSFC and RAG1KOnoAT. Blue dots represent downregulated SFs, and red dots represent upregulated SFs. Notably, 0 SFs were either up- or downregulated in the comparison between RAG1KOATSFL and RAG1KOATSFC. **(B)** The number of alternative splicing events across five major categories (explained in the left graph) changed following CD4^+^ T-cell transfer across group comparisons. **(C)** Genes with RNA splicing events affected by Prob-SF-CD4^+^ T-cell transfer that are associated with the inflammatory network. Irf7, Irak1, and Map3k7, shown as square-shaped nodes, are genes with RNA splicing. RNA splicing categories are indicated within the squares in green. Irf7 shows SE, RI, and MXE events; Irak1 shows SE and A3SS events; and Map3k7 shows only SE RNA splicing (*n* = 3 mice per group).

Alternative RNA splicing is a vital process through which different protein variants can be produced from a single gene. Aberrant RNA splicing can lead to dysfunctional proteins and contribute to various autoimmune disorders, including autoimmune hepatitis (58). Many splicing factors (SFs) have been identified that are recruited to the spliceosome at specific stages of the splicing cycle (59). We found that AT of SF-CD4^+^ T cells to RAG1KO mice altered SF expression. Among the DEGs, 26 SFs were downregulated, and 10 SFs were upregulated (**Figure 6A**). These SFs participate in RNA splicing events, leading to a complex interplay with inflammatory processes. For example, five representative SFs upregulated by SF-CD4^+^ T cells—including *Lgals3* (encodes galectin-3), *Slc38a2* (also called SNAT2, a sodium-dependent neutral amino acid transporter), small nuclear ribonucleoprotein 70 (*Snrnp70*), immunoglobulin-like domain containing receptor 2 (*Ildr2*), and RNA-binding motif 25 (*Rbm25*)—mediate RNA splicing events that generally promote inflammatory processes. In contrast, four representative SFs downregulated by SF-CD4^+^ T cells—splicing factor 3b subunit 6 (*Sf3b6*), pre-mRNA processing factor 38A (*Prpf38a*), THO complex subunit 7 (Thoc7), and aldo-keto reductase family 1, member C6 (*Akr1c6*)—are considered negative regulators of NF-κB signaling and/or organizers for protein assembly and function (60–63).

RNA splicing events across five major categories were analyzed, as shown in **Figure 6B** (left graphic panel), including the identification of the number of exon inclusion (Inc) and exon exclusion (Exc) events. We found that the number of categorized RNA splicing events—SE (Inc and Exc), A3SS (Exc), MXE (Exc), and RI (Exc)—as well as total RNA splicing events (Inc and Exc), was reduced in DEGs of RAG1KOATSFL vs. RAG1KOATSFC, compared with DEGs of RAG1KOATSFC vs. RAG1KOnoAT (**Figure 6B**, right table). These results indicate that Prob-SF-CD4^+^ T cells can reduce the number of RNA splicing events induced by SF-CD4^+^ T cells.

We further explored which genes undergo RNA splicing events affected by Prob-SF-CD4^+^ T cells. We identified three genes—interferon regulatory factor 7 (*Irf7*), interleukin-1 receptor-associated kinase 1 (*Irak1*), and *Map3k7* (also known as TGF-β-activated kinase [TAK1])—which underwent SE, RI, and MXE for *Irf7*; SE and A3SS for *Irak1*; and RI for *Map3k7*, with multiple time splicing events over time (**Figure 6C**). These three genes are associated with the inflammatory gene interactive network and have the ability to interact with NF-κB or MAPK signaling to promote inflammation. RNA splicing of these genes, as affected by Prob-SF-CD4^+^ T cells, may generate different isoforms that act as negative regulators of inflammation (64).

### 3.6 Adoptive transfer of CD4^+^ T cells from SF mice to RAG1KO altered gut microbiota

The immune system and gut microbiota have a complex, interconnected relationship. The gut microbiota interacts with the immune system through metabolites, by stimulating immune cells, and thereby modulating immune responses (65). We investigated whether AT of SF-CD4^+^ T cells or Prob-SF-CD4^+^ T cells could shape the gut microbiota and influence its composition.

**Figure 7:**
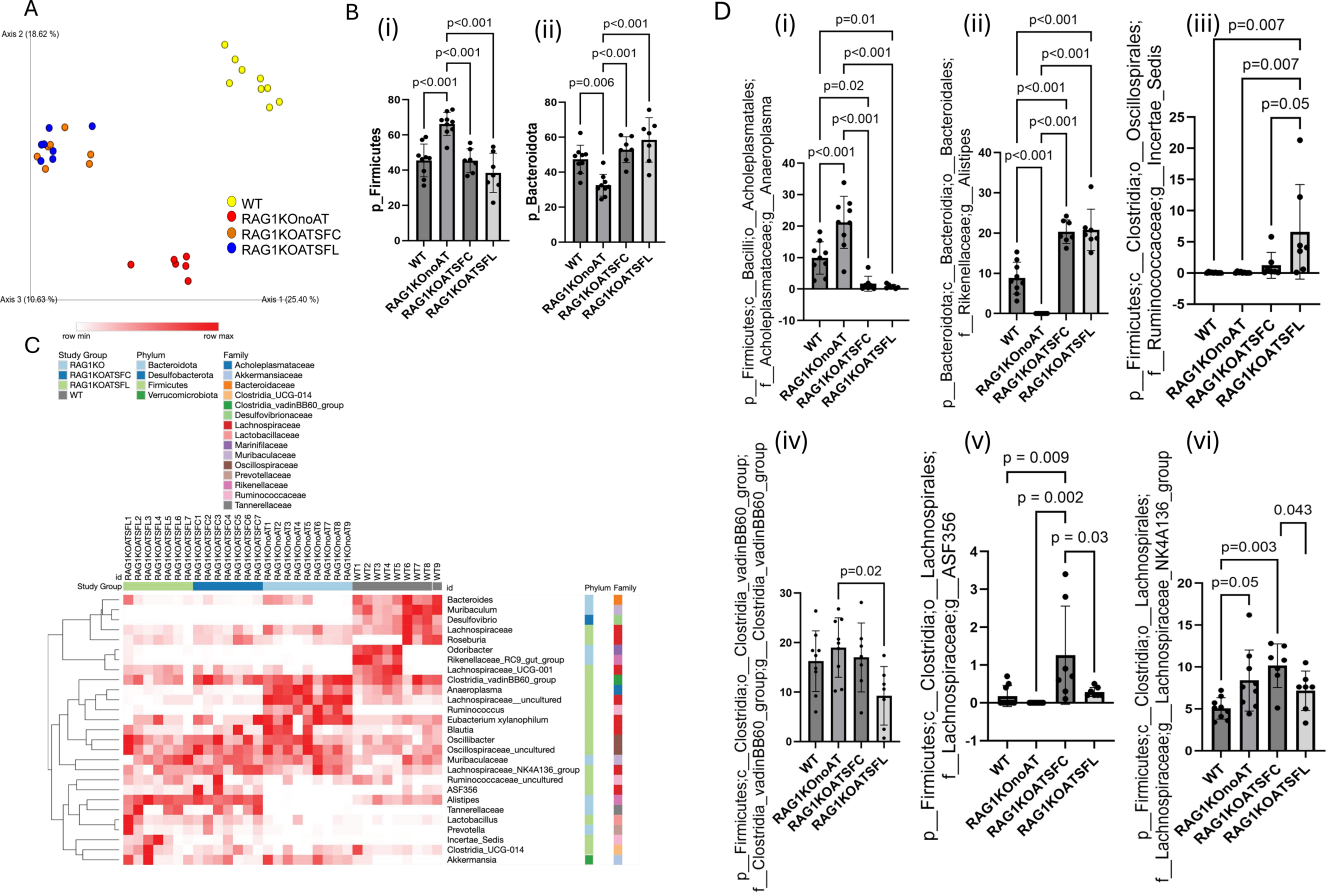
Gut microbiota analysis in RAG1KO mice after adoptive transfer of CD4^+^ T cells. **(A)** Bray–Curtis beta-diversity. **(B)** Relative abundance of phylum-level Firmicutes (i) and Bacteroidota (ii) across different groups. **(C)** Heatmap displaying the relative abundances of bacterial composition (phylum–family–genus levels) across the four groups: WT, RAG1KOnoAT, RAG1KOATSFC, and RAG1KOATSFL. **(D)** Relative abundance at the genus level of *Anaeroplasma* (i), *Alistipes* (ii), *Incertae_sedis* (iii), *Clostridia_vadinBB60_group* (iv), *ASF356* (v), and *Lachnospiraceae_NK4A136_group* (vi), compared across the different groups. Significant *p*-values (*p* < 0.05) are indicated in the figures (*n* = 7–9 mice per group).

Microbiome analysis of RAG1KO mice following AT SF-CD4^+^ T cells (RAG1KOATSFC) revealed a shift in microbial clusters compared with RAG1KO mice without T-cell transfer (RAG1KOnoAT). The microbial cluster of AT of Prob-SF-CD4^+^ T cells in RAG1KO mice (RAG1KOATSFL) grouped with RAG1KOATSFC (**Figure 7A**). Altered α-diversity was associated with CD4^+^ T-cell transfer, regardless of whether CD4^+^ T cells were modulated by probiotics or not (**Supplementary Figure S6**). The abundance of certain bacterial taxa was altered by AT of SF-CD4^+^ T cells, but these were also altered by AT of Prob-SF-CD4^+^ T cells, with no significant differences between the CD4^+^ T-cell groups. At the phylum level, Firmicutes and Bacteroidota predominated in WT stool, comprising ~ 40%–50% of relative abundance (RA) (**Figure 7B**). The RA of Firmicutes was increased in untreated RAG1KOnoAT mice compared with WT mice (*p* < 0.001) and was significantly decreased by AT of SF-CD4^+^ T (*p* < 0.001) or Prob-SF-CD4^+^ T cells (*p* < 0.001) (**Figure 7B**i). In contrast, the reduced RA of Bacteroidota in RAG1KO mice compared with WT (*p* = 0.006) was reversed in mice receiving AT of SF-CD4^+^ T cells (*p* < 0.001) or Prob-SF-CD4^+^ T cells (*p* < 0.001) (**Figure 7B**ii). The heatmap shows that cluster bacteria in WT stool are distinct from those in RAG1KOnoAT, whereas adoptive transfer of RAG1KO altered the cluster bacteria observed in RAG1KOnoAT (**Figure 7C**). Specifically, at the genus level, the RA of *Anaeroplasma* was increased in RAG1KOnoAT mice compared with WT (*p* < 0.001) and was significantly reduced by SF-CD4^+^ T^-^cell transfer, regardless of whether the cells had undergone probiotic modification (**Figure 7D**i). *Alistipes*, which are depleted in RAG1KOnoAT mice compared with WT (*p* < 0.001), were significantly increased by SF-CD4^+^ T cells, without being influenced by probiotic modification (**Figure 7D**ii). Interestingly, we noted that the RA of *Incertae_Sedis* approached a significant increase (*p* = 0.05; **Figure 7D**iii), while *Clostridia_vadinBB60_group* (**Figure 7D**iv), *ASF356* (**Figure 7D**v), and *lachnospiraceae_NK4A136_group* (**Figure 7D**vi) were significantly decreased following the transfer of Prob-SF-CD4^+^ T cells compared with SF-CD4^+^ T cells. The RA of *Lachnospiraceae_uncultured* (**Supplementary Figure S7**iv) and *Eubacterium xylanophilum* (**Supplementary Figure S7**v) was increased in RAG1KOnoAT mice compared with WT mice but reduced by CD4^+^ T-cell transfer. In contrast, the RA of *Tannerellaceae* (**Supplementary Figure S7**vii) and *Lactobacillus* (**Supplementary Figure S7**viii) increased following CD4^+^ T-cell transfer. Mouse stool samples contained a relatively higher abundance of *Muribaculaceae* (~ 20%–40%) with no changes among the groups (**Supplementary Figure S7**i). These findings indicate that, although the transfer of probiotic-modulated SF-CD4^+^ T cells has minimal overall effects on gut microbiota compared with SF-CD4^+^ T cells in immunocompromised mice, prob-SF-CD4^+^ T cells may distinctly influence certain bacterial genera, such as *Incertae_Sedis* and the *Clostridia_vadinBB60_group.*

## 4 Discussion

This study characterized hepatic changes resulting from the transfer of CD4^+^ T cells isolated from Treg-deficient SF mice (SF-CD4^+^ T cells) and from SF mice orally administered with *L. reuteri* DSM 17938 (Prob-SF-CD4^+^ T cells) into immunodeficient hosts (T- and B-cell-deficient RAG1KO mice). We show that adoptive transfer of SF-CD4^+^ T or Prob-SF-CD4^+^ T cells modulates liver inflammation and the expression of liver genes associated with MΦ polarization, inflammation, and metabolism. Furthermore, CD4^+^ T^-^cell transfer induced RNA splicing events and modified gut microbiota composition. These findings indicate that CD4^+^ T cells engage in complex crosstalk with MΦs, cell organelles (e.g., mitochondria), and signaling pathways within the liver.

Adoptive transfer of SF lymphocytes, isolated from the spleen or lymph nodes, has been shown to drive autoimmunity and multiorgan inflammation in RAG1KO mice (17, 66, 67). SF mice may present a broad functional autoimmune repertoire, as even small numbers of CD4^+^ T cells—2 × 10^5^ cells transferred by intramuscular (IM) injection or 1.25 × 10^6^ cells transferred by intravenous (IV) or IP injection—are sufficient to induce multiorgan inflammation (67). In the current study, we found that IP transfer of only 1 × 10^6^ splenic CD4^+^ T cells was sufficient to induce liver inflammation.

MΦs are critical components of the liver’s immune system, maintaining homeostasis and regulating injury response. In AIH, the imbalance between MΦ subsets M1 and M2 is considered a key factor in AIH development (36, 68). SF-CD4^+^ T-cell transfer not only increased the numbers of MΦ in the liver of RAG1KO mice but also upregulated genes involved in MΦ polarization that could be downregulated by Pro-CD4^+^ T-cell transfer. Interestingly, altered genes were involved in almost equal associations with M1 and/or M2 polarization and differentiation, indicating complex CD4^+^ T-cell–MΦ interactions. Some genes associated with M2, such as SH2 domain-containing adaptor protein B (Shb), do not significantly impact M1, but their *deficiency* biases MΦ toward the M2 phenotype (69). MΦs are highly plastic cells that exist on a continuum of activation states. Shared genes may be associated with both M1 and M2 (70, 71), and their specific function and expression levels depend on signals from the local microenvironment, creating a diverse range of overlapping phenotypes.

SF-CD4^+^ T cells upregulated genes associated with inflammatory TLR cascades/MARK pathway linked with cytokine signaling. These genes directly or indirectly control each other’s activity within a gene interaction network, a pattern that was largely reversed by Prob-SF-CD4^+^ T cells. TLRs constitute the forefront of the innate immune system and, in response to a ligand, can activate downstream cascades of inflammatory pathways, including the release of proinflammatory cytokines (72). We noticed that Prob-SF-CD4^+^ T cells specifically modulated five regulatory genes in TLR/NF-κB signaling, including Tank, *Cul1*, *Fbxw11*, *Ubc*, and *Ppp2ca*, suggesting a distinct role in modulating this signaling to maintain immune homeostasis and prevent autoimmune diseases. For example, the Tank gene influences both NF-κB and IFN responses, acts as both a positive and negative regulator, and participates in crosstalk between different signaling pathways (73). Cul1, Fbxw11, and Ubc play important regulatory roles in fine-tuning TLR-mediated innate immune responses through the ubiquitin–proteasome system, controlling the intensity and duration of immune responses. Dysregulation of Cul1 activity or its associated complexes could have significant consequences, potentially contributing to the development of autoimmune diseases or exacerbating inflammatory conditions (74); Fbxw11 functions in NF-κB pathway activation and has been identified as a novel inflammatory biomarker in pancreatitis and pancreatic cancer (75). Although Ppp2ca often acts as a negative regulator of TLR signaling by dephosphorylating MyD88 (76), variations in this gene have been linked to susceptibility to autoimmune diseases such as systemic lupus erythematosus (SLE), with higher serum levels of IL-6 and IL-17 (77). The MAPK signaling pathway transmits extracellular signals to the nucleus and becomes activated during inflammation, leading to the production of inflammatory molecules. It is recognized as a crucial signaling hub in AIH, activated by both T cells and liver cells, thereby driving intense liver inflammation, the release of cytokines such as IL-6, TNF-α, and IFN-γ, and subsequent liver damage (78).

Importantly, Lcn2 was downregulated by Prob-SF-CD4^+^ T cells compared with SF-CD4^+^ T cells. Lcn2 is a multifaceted innate immune protein, often released by neutrophils, suggesting a CD4^+^ T-cell–neutrophil interaction. Lcn2 plays dual roles in inflammation: it helps fight bacterial infections by sequestering iron to limit bacterial growth, but it can also recruit neutrophils and promote inflammation in conditions including autoimmune diseases. The study has shown that Lcn2 can enhance probiotic survival and efficacy in inflamed guts, whereas dysbiosis is linked to high Lcn2 levels, which reduced beneficial bacteria, highlighting a complex interplay in which probiotics might help balance the proinflammatory effects of Lcn2 (55). Regulation of TLR signaling, neutrophils, and inflammatory pathways is considered a therapeutic target for AIH (79–81).

Interestingly, we observed that the Dectin pathway was upregulated by SF-CD4^+^ T-cell transfer compared with absent CD4^+^ T-cell transfer, whereas it was downregulated by Prob-SF-CD4^+^ T-cell administration compared with SF-CD4^+^ T-cell transfer. Dectin-1, a C-type lectin receptor, normally recognizes fungal components but also interacts with endogenous molecules in the liver, enabling modulation of innate immune responses even in the absence of infection. The Dectin plays dual roles in regulating liver inflammation. For example, it can protect against liver inflammation by dampening TLR4 signaling during hepatic fibrosis (51) or, conversely, contribute to nonalcoholic steatohepatitis (NASH) when Dectin receptors are overexpressed and recognize liver-released pathogenic molecules (82).

SF-CD4^+^ T cells were found to exert a significant impact on mitochondria due to the downregulation of genes, such as those in the MRP and RPS families, which are crucial for mitoribosome assembly and the maintenance of mitochondrial structure and function (83). The gene downregulation induced by SF-CD4^+^ T cells also affects MRC complexes I–V, leading to increased oxidative stress. MRC dysfunction and the subsequent disruption of energy production are pivotal in AILD, such as AIH and PBC (43).

Members of the Ugt2b gene family encode UDP-glucuronosyltransferase (UGT) enzymes, such as Ugt2b38 and Ugt2b37, which are highly altered by SF-CD4^+^ T cells. These enzymes catalyze glucuronidation, a process in which a glucuronic acid molecule is added to various compounds (toxins and drugs), increasing their water solubility and allowing more efficient excretion from the body via urine or bile (84). Previous studies reported that Ugt2b38 expression is male-predominant in the kidney, but not the liver, and its regulation is linked to testosterone levels in C57BL/6 mice (85). However, both RNAseq and qRT-PCR analyses indicated that the *Ugt2b38* gene is expressed in the liver of RAG1KO mice and is downregulated by SF-CD4^+^ T-cell transfer.

We noticed that mouse Mup genes were downregulated by SF-CD4^+^ T cells in the liver of RAG1KO mice. The Mup family comprises at least 21 functional low-molecular-weight (18–19 kDa) isoforms, which are predominantly expressed in the liver in a sexually dimorphic pattern and excreted in the urine of males, where they act as pheromones for social communication and individual identification. Deletion of the Mup gene cluster results in metabolic shifts and lipid accumulation (86). The role of Mup genes in autoimmune liver diseases requires further investigation.

As shown in our study, SF-CD4^+^ T cells upregulated inflammatory pathways in the liver of RAG1KO mice compared with RAG1KO mice without CD4^+^ T-cell transfer. In contrast, transferring Prob-SF-CD4^+^ T cells vs. SF-CD4^+^ T cells downregulated inflammatory pathways. SF-CD4^+^ T-cell transfer also downregulated genes related to metabolism, including the TCA cycle, lipid and organic compound levels, and liver detoxification, compared with their expression in RAG1KO mice without CD4^+^ T-cell transfer. Conversely, Prob-SF-CD4^+^ T cells upregulated mitochondrial pathways, the TCA cycle, and cell-cycle-related genes compared with SF-CD4^+^ T cells. Future studies should determine whether these pathways are modulated by probiotics in SF mice and identify the distinct metabolic signatures that shape their CD4^+^ T-cell phenotype. Metabolite influences on CD4^+^ T cells are crucial but complex. We previously showed that DSM 17938 converts ATP and AMP to adenosine because the strain uniquely contains a gene encoding a protein with ecto-5′-nucleotidase (5NT) activity (87). Plasma adenosine or inosine levels were lower in SF mice compared with WT control mice, a condition that was reversed by DSM 17938 treatment but not by the DSM 17938 strain carrying a 5NT gene mutation (87). Adenosine and inosine function as anti-inflammatory nucleosides by binding A2a receptors on inflammatory T cells (Th1/Th2), thereby inhibiting Th1/Th2 responses (3, 14). Adenosine also modulates immune balance by shifting the Treg/Th17-cell ratio in favor of Treg cells. Recent studies showed that although the adenosine agonist 5-*N*-ethyl-carboxamide adenosine (NECA) promotes expansion of IL-17^+^CD4^+^ T cells during T-cell activation, these Th17 cells display a noninflammatory cytokine and gene expression profile (88). Adenosine can directly inhibit the induction of cytokines IL-2, IFN-γ, TNF-α, and IL-4 in peripheral lymphocytes, thereby modulating T cells and further dampening inflammation (89). Clarifying the metabolite pathways, gene signatures, and mechanisms by which DSM 17938 “educates” CD4^+^ T cells in the liver microenvironment will be beneficial for future probiotic-based therapeutic strategies.

RNA splicing is a crucial step in gene expression, in which noncoding regions (introns) are removed from RNA transcripts and coding regions (exons) are joined to produce a mature mRNA molecule (90, 91). However, altered proteins can result in dysfunctional proteins, leading to cellular dysfunction and disease. CD4^+^ T cells have been shown to influence RNA splicing in the liver, particularly in inflammatory environments where they become activated and infiltrate hepatic tissue. The proinflammatory milieu includes increased expression and activity of SFs, which alter the RNA splicing landscape and may create a feedback loop that promotes disease progression in the liver (92, 93). In our study, proinflammatory SF-CD4^+^ T cells affected SFs in DEGs; for example, Lgals3, which encodes galectin-3, is known to bind to carbohydrates and interact with RNA molecules. In fact, Snrnp70, Rbm25, Sf3b6, and Prpf38a are all involved in mRNA splicing via the spliceosome. SF-CD4^+^ T-cell transfer not only altered SFs but also affected the number of RNA splicing events, whereas Prob-SF-CD4^+^ T cells reduced events primarily associated with exon exclusion in the livers of RAG1KO mice. Genes undergoing RNA splicing are part of the TLR (innate immunity) networks, suggesting that CD4^+^ T-cell transfer influences RNA splicing, which may modulate liver inflammation.

The fecal microbial community in CD4^+^ T-cell-treated RAG1KO mice became significantly distinct from that of RAG1KOnoAT mice. We found that the RAs of phylum Firmicutes, genus *Anaeroplasma*, were reduced, whereas those of phylum Bacteroidetes, genus *Alistipes*, were increased compared with RAG1KOnoAT mice. We hypothesize that CD4^+^ T cells could alter the gut microbiota through a variety of mechanisms, such as producing cytokines that influence bacterial growth, directly interacting with gut microbes, and/or indirectly modulating immune responses to resident microbes in the gut (94).

Changes of bacterial diversity following CD4^+^ T-cell transfer were not affected by T cells modulated through probiotic feeding. The results indicate that (i) AT of SF-CD4^+^ T cells significantly impacts gut microbiota, and (ii) AT of Prob-SF-CD4^+^ T-cells has minimal effects on the gut microbiota in immunocompromised RAG1KO mice. AT of CD4^+^ T cells from healthy donors is known to maintain gut homeostasis, promote “mutualism” with beneficial microbes, and prevent inflammation (95). Despite no overall change in diversity, Prob-SF-CD4^+^ T-cell transfer increased the RA of *Incertae_sedis* and reduced the RA of *Clostridia_vadinBB60_group*, *ASF356*, and *Lachnospiraceae NK4A136* group in the stool. 

The RA of *Anaeroplasma* in multiple sclerosis has been positively correlated with disease severity, and this taxon could be reduced by DSM 17398 (96, 97). ASF356 is a specific strain of *Clostridium* bacteria within a defined microbial community, the Altered Schaedler Flora (ASF), used in research. ASF356 contributes to vascular aging in the host through its production of the inflammatory metabolite phenylacetic acid (PAA), which promotes endothelial cell senescence and dysfunction by increasing oxidative stress and a senescence-associated secretory phenotype (SASP) (98). The *Lachnospiraceae NK4A136* group has a dual relationship with inflammatory cells, potentially exerting both protective and detrimental effects depending on the context. Its production of anti-inflammatory SCFAs, including butyrate, can be protective (99); however, strain *NK4A136* has also been linked to inflammation in certain disease models (100). In contrast, *Alistipes*, a beneficial microbiome of the *Rikenellaceae* family, is depleted in patients with liver cirrhosis, nonalcoholic fatty liver disease (NAFLD), and hepatocellular carcinoma (HCC) (101). *Incertae sedis* is a Latin term meaning “of uncertain placement”, used in biological classification for a taxonomic group whose broader relationships to other groups are unknown or undefined. Recent studies have shown that it plays a role in the fermentation of dietary fibers and the production of SCFAs (102) and has a protective effect in chronic liver disease (103). Additionally, *Clostridiales vadin BB60_ grou*p has been reported to have a potential causal relationship with AILD (104) and has been identified as a risk factor for Graves’ disease (GD) (105).

In conclusion, our study shows that lymphocytes (CD4^+^ T cells) isolated from a host with autoimmune inflammation can evoke an exaggerated immune response in the liver of an immunologically naïve host. However, when the diseased host was treated with probiotic *L. reuteri* DSM 17938, the transferable probiotic-exposed lymphocytes produced transcriptional shifts in liver tissue that could benefit the recipient. This benefit resulted from the activation of multiple genes, RNA splicing events, and gene interactions. Further mechanistic studies are necessary to elucidate how probiotics educate inflammatory CD4^+^ T cells. This research could identify new options for treating AILD and other autoimmune disorders.

## Data Availability

The datasets of stool microbiota sequencing presented in this study can be found in the National Center for Biotechnology Information (NCBI)/Sequence Read Archive (SRA)[BioProject ID: PRJNA1310988]. The datasets of liver RNAseq presented in this study can be found in the NCBI/SRA [BioProject ID:PRJNA1359415]. The original contributions presented in the study are included in the article and Supplementary Material. Further inquiries can be directed at the corresponding author.
