# Supplementary material for: Limosilactobacillus reuteri alleviates proinflammatory T-cell-mediated liver injury and transcriptomic changes in immunocompromised mice
Source: Front Immunol. 2026 Mar 3;17:1713120. doi: 10.3389/fimmu.2026.1713120 (PMC12992261; doi:10.3389/fimmu.2026.1713120)
Supplement: Supplementary file 1 [file DataSheet1.pdf]

Supplementary Materials

Supplementary Table 1. The number of mice in each batch of experiments and their samples collected for analysis

Batch of experiment	#1	#2	#3	#4	#5
Mice (number of mice)	WT (n=5)<br>RAG1KOnoAT (n=5)	WT (n=4)<br>RAG1KOnoAT (n=4)	RAG1KOATSFC (n=3)<br>RAG1KOATSFL (n=3)	RAG1KOATSFC (n=3)<br>RAG1KOAT SFL (n=4)	RAG1KOATSFC (n=4)<br>RAG1KOATSFL (n=3)
Biospecimens collected	Stool	Stool<br>Liver histology<br>Liver frozen	Liver histology	Stool<br>Liver histology<br>Liver frozen	Stool<br>Liver histology<br>Liver frozen

For liver histological analysis, the number of mice in each group: WT n=4; RAG1KOnoAT n=4; RAG1KOATSFC n=10, RAG1KOATSFL n=10 (Figure 2, Figure 3AB).

For stool microbiota analysis, the number of mice in each group: WT n=9, RAG1KOnoAT n=9, RAG1KOATSFC n=7, RAG1KOATSFL n=7 (Figure 7; Supplementary Figure 4 and Supplementary Figure 5).

For liver RNAseq analysis, the number of mice in each group for initially isolating RNAs from fresh frozen liver tissues: RAG1KOnoAT n=4, RAG1KOATSFC n=7, RAG1KOATSFL n=7; the number of mice in each group with high quality RNAs (RNA Integrity Number >7) used for RNA sequencing: RAG1KOnoAT n=4, RAG1KOATSFC n=6, RAG1KOATSFL n=6; the number of mice in each group with high quality raw sequencing reads after being evaluated using FastQC and summarized with MultiQC and eventually were used for analyzing differentially expressed genes (DEGs), pathways, and RNA diversity: RAG1KOnoAT n=3, RAG1KOATSFC n=3, RAG1KOATSFL n=3 (Figure 3C, Figure 4, Figure 5, Figure 6; Supplementary Figure 1 and Supplementary Figure 2; Supplementary Table 2 and Supplementary Table 4).

For RT-qPCR gene verification: RAG1KOnoAT n=4, RAG1KOATSFC n=6, RAG1KOATSFL n=6 (Supplementary Figure 3).

Supplementary** Figure 1. **Gating strategy to identify purity of CD4$^+$T cells comparing before and after cell isolation

The purity of CD4$^+$T cells >90%, non-CD4$^+$T cells (CD4$^-$cells) and naïve-like-non-CD4$^+$T cells (CD4$^-$CD45RB^{high}) have been removed from splenocytes of SF mice by flow cytometry analysis

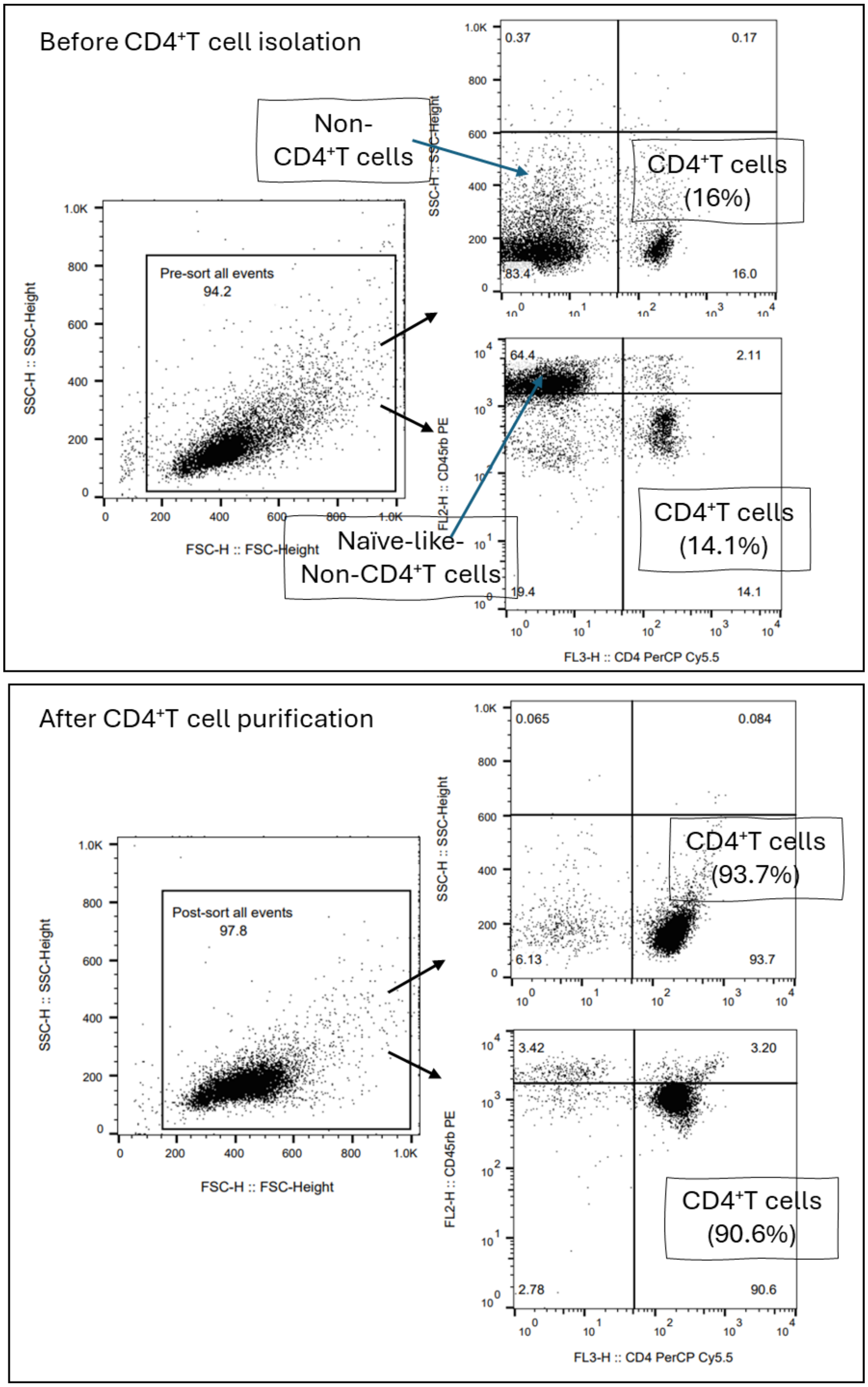

Supplementary** Figure 2**. Per-base mean quality scores across all RNA-seq libraries

All samples (n=3/group) displayed uniformly high per-base sequence quality, with Phred scores exceeding 33 across all positions

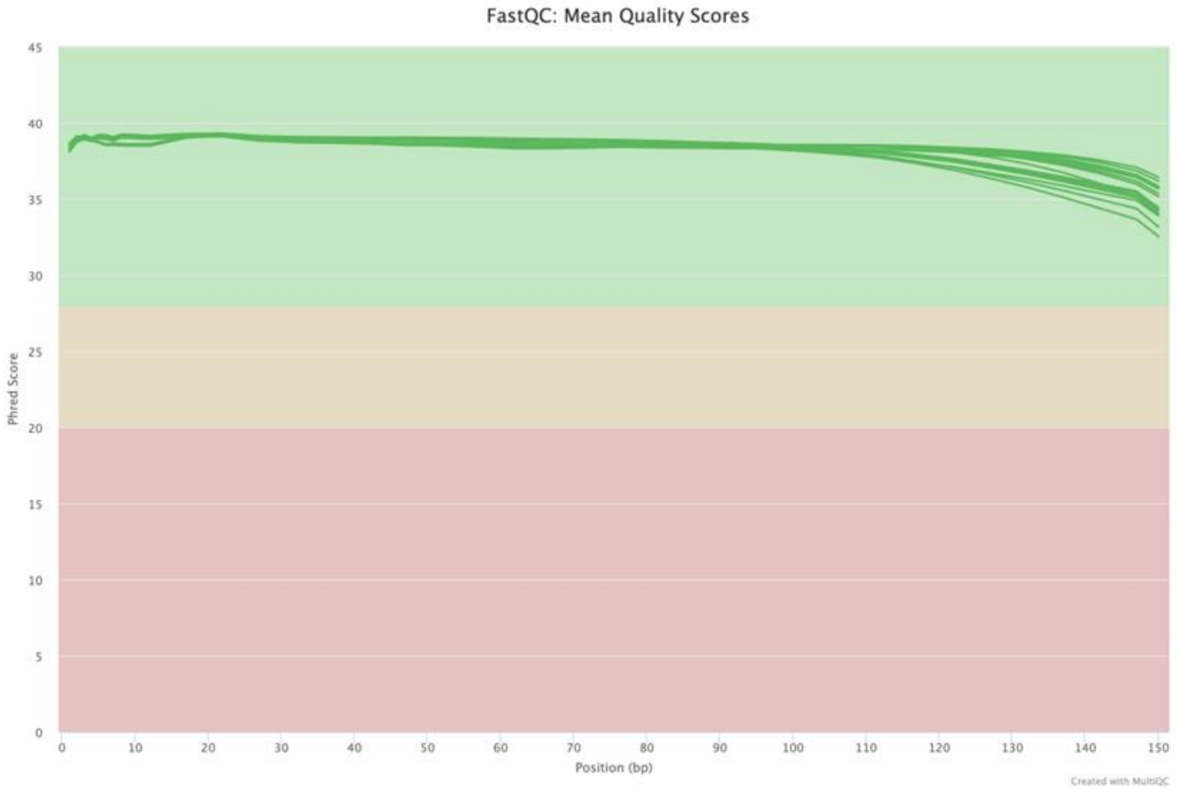

Supplementary** Table 2. **Alignment and Mapping Summary for RNA-seq Libraries

Study Group	Sample Name	Number of input reads	Total Mapped
RAG1KOnoAT	RAG1KOnoAT_Rep1	38514713	75.45%
	RAG1KOnoAT_Rep2	38119389	73.34%
	RAG1KOnoAT_Rep3	38313189	82.24%
RAG1KOATSFC	RAG1KOATSFC_Rep1	39109596	93.40%
	RAG1KOATSFC_Rep2	45579561	91.70%
	RAG1KOATSFC_Rep3	43606057	87.02%
RAG1KOATSFL	RAG1KOATSFL_Rep1	37130488	81.58%
	RAG1KOATSFL_Rep2	44534269	68.50%
	RAG1KOATSFL_Rep3	44409887	93.23%

Supplementary** Figure 3**. PCA using normalized gene counts to assess global expression variation and sample relationships

Top: : PCA of RAG1KOATSFL and RAG1KOATSFC Samples
Bottom: PCA of RAG1KOATSFC and RAG1KOnoAT Samples

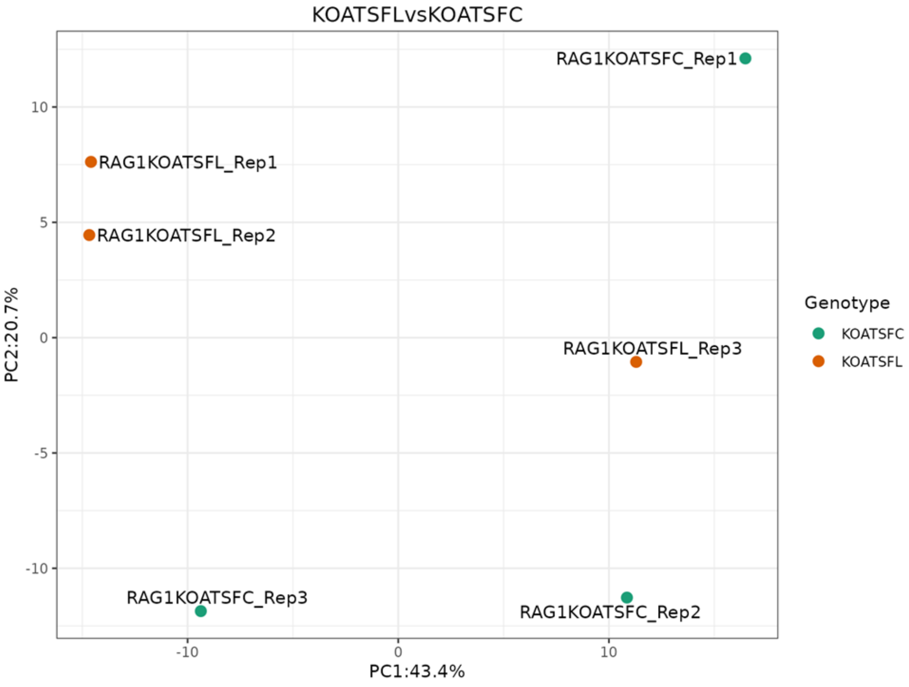

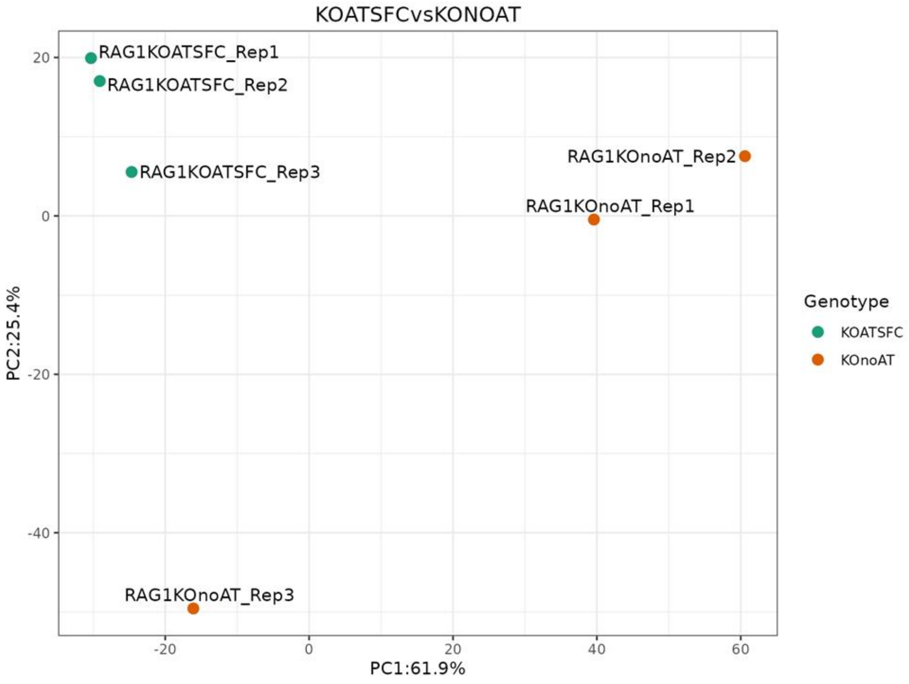

Supplementary** Figure 4. **Verification of gene expressions in the liver of RAG1KO mice with or without adoptive transfer (AT) of SF-CD4^{+}T cells was performed using qRT-PCR

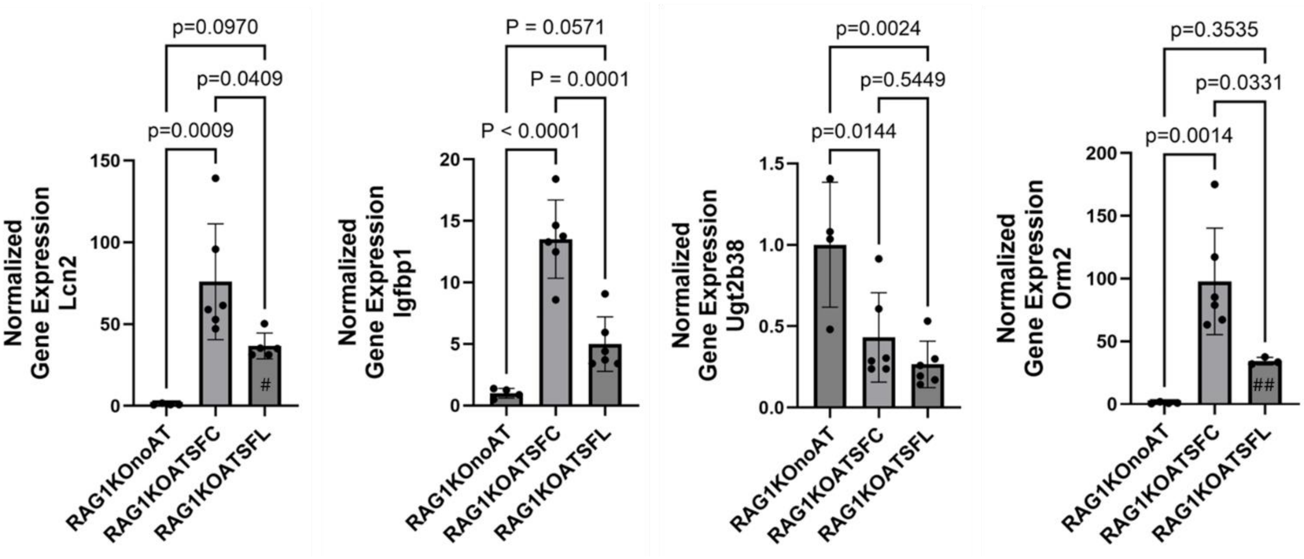

n=5 excluded one sample that was not used for RNAseq final analysis
n=3 included three samples used for RNAseq final analysis

Supplementary** Table 3. **Primers used for qRT-PCR

**Primers**	**Forward**	**Reverse**
Lcn2	TGGCCCTGAGTGTCATGTG	CTCTTGTAGCTCATAGATGGTGC
Igfbp1	ATCAGCCCATCCTGTGGAAC	TGCAGCTAATCTCTCTAGCACTT
Ugt2b38	TGCGCCACAAAAGGGCTAA	ACACAAGAGAGTAGGAAGCCG
Orm2	GGCGACCCTATCACCAATGA	TTAGGACAGCCGCACCAATG

Supplementary** Table 4. **Genes affect macrophage polarization and metabolism that are upregulated by SF-CD4^{+}T cells but reversed by Prob-SF-CD4^{+}T cells in the liver of recipient RAG1KO mice

*Highlighted genes are associated with MΦ metabolism: yellow-highlighted genes regulate lipid metabolism, light-blue highlighted genes regulate iron metabolism, green-highlighted gene regulates glucose homeostasis, and light-grey-highlighted genes regulate amino acid physiology.

Gene symbol	Ensembl ID	Full Name (Encodes protein)	MΦ subtypes	Liver Metabolism (MΦ Metabolism)	Refs
Apol11b	ENSMUSG00000091694.9	Apolipoprotein L 11b	M1	Lipid (Lipid, Polyamine)	(1-3)
Apcs	ENSMUSG00000026542.6	Serum amyloid P component	M1	-	(4, 5)
CD53	ENSMUSG00000040747.9	CD53 (tetraspanin web)	M1	Lipid (Lipid)	(6-8)
CP	ENSMUSG00000003617.16	Ceruloplasmin	M1	Iron, Lipid, Bile Acid (Iron)	(9-11)
Ctsc	ENSMUSG00000030560.17	Cathepsin C	M1	Lipid (Fatty Acid)	(12-14)
Gadd45g	ENSMUSG00000021453.2	Growth arrest DNA damage-inducible gene 45	M1	-	(15)
Hck	ENSMUSG00000003283.14	Hematopoietic cell kinase	M1	-	(16)
Lcn2	ENSMUSG00000026822.14	Lipocalin-2	M1	Lipid (Iron)	(17-21)
Ly86	ENSMUSG00000021423.6	Myeloid differentiation protein 1	M1	Lipid (Lipid)	(22)
Orm2	ENSMUSG00000061540.3	Orosomucoid-2	M1	Lipid, Bile Acid	(23, 24)
P2ry13	ENSMUSG00000036362.2	purinergic receptor P2RY13	M1	Lipid	(25, 26)
Serpia3N	ENSMUSG00000021091.8	Serine peptidase inhibitor, clade A, member 3N, α-1-antichymotrypsin	M1	Lipid	(27, 28)
CD163	ENSMUSG00000008845.9	CD163	M2	Lipid, Glucose (Heme/Iron)	(29, 30)
Cxcl14	ENSMUSG00000021508.11	Chemokine (C-X-C motif) ligand 14	M2	Lipid	(31, 32)
Fcgr3	ENSMUSG00000059498.13	Fc gamma receptor III	M2	-	(33)
Hmox1	ENSMUSG00000005413.8	Heme oxygenase-1 (HO-1)	M2	Heme/Iron (Iron)	(34, 35)
Irf2bp2	ENSMUSG00000051495.7	(Interferon Regulatory Factor 2 Binding Protein 2)	M2	Lipid (Lipid)	(36, 37)
Igfbp1	ENSMUSG00000020429.7	Insulin-like growth factor-binding protein 1	M2	Lipid, Glucose (Lipid)	(38-41)
Itih3	ENSMUSG00000006522.17	Inter-alpha-trypsin inhibitor heavy chain (ITIH) 3	M2	Lipid	(42, 43)
Itih4	ENSMUSG00000021922.17	Inter-alpha-trypsin inhibitor heavy chain (ITIH) 4	M2	Lipid	(44, 45)
Mafb	ENSMUSG00000074622.4	MAF basic region leucine zipper (bZIP) transcription factor B	M2	Lipid	(46, 47)
Orm1	ENSMUSG00000039196.2	Orosomucoid-1	M2	Lipid, Glucose	(48, 49)
Prtn3	ENSMUSG00000057729.12	proteinase 3	M2	Lipid	(50, 51)

Shb	ENSMUSG00000044813.15	SH2 domain-containing adaptor protein B	M2	Lipid	(52, 53)
Slc7a8	ENSMUSG00000022180.7	Solute Carrier Family 7 Member 8; Large neutral amino acids transporter small subunit 2 (LAT2)	M2	Amino Acid (Amino Acid)	(54, 55)
Slc38a2	ENSMUSG00000022462.7	Solute carrier protein 38	M2	Amino Acid (Glutamine)	(56, 57)
Spp1	ENSMUSG00000029304.14	Secreted phosphoprotein 1(Osteopontin)	M2	Lipid (Lipid)	(58, 59)
Vsig4	ENSMUSG00000044206.3	V-set and immunoglobulin domain containing 4 B7-family related protein	M2	Amino Acid (Pyruvate)	(60, 61)
Ahr	ENSMUSG00000019256.17	Aryl hydrocarbon Receptor	M1 and M2	Lipid (Lipid)	(62, 63)
CD68	ENSMUSG00000018774.13	CD68	M1 and M2	Lipid (Lipid)	(64, 65)
Irs2	ENSMUSG00000038894.7	Insulin Receptor Substrate-2	M1 and M2	Glucose (Glucose)	(66, 67)
Saa1	ENSMUSG00000074115.5	Serum amyloid A1	M1 and M2	Lipid	(68-71)
Saa2	ENSMUSG00000057465.5	Serum amyloid A2	M1 and M2	Lipid	(70, 71)
Saa4	ENSMUSG00000040017.8	Serum amyloid A4	M1 and M2	Lipid	(70-72)
Slc25a25	ENSMUSG00000026819.15	solute carrier protein 25	M1 and M2	Glucose (Cholesterol)	(55, 73, 74)
Ucp2	ENSMUSG00000033685.13	Uncoupling protein 2	M1 and M2	-	(75)

References

1. Ryu JH, Ge M, Merscher S, Rosenberg AZ, Desante M, Roshanravan H, et al. Apol1 Renal Risk Variants Promote Cholesterol Accumulation in Tissues and Cultured Macrophages from Apol1 Transgenic Mice. *PLoS One* (2019) 14(4):e0211559. Epub 20190418. doi: 10.1371/journal.pone.0211559.

2. Shen X, Chen M, Zhang J, Lin Y, Gao X, Tu J, et al. Unveiling the Impact of Apof Deficiency on Liver and Lipid Metabolism: Insights from Transcriptome-Wide M6a Methylome Analysis in Mice. *Genes* (2024) 15(3):347.

3. Liu E, Wright M, Kearney AO, Caza T, Yang JY, Garcia V, et al. *G1 and G2 Apolipoproteinl1 Modulate Macrophage Inflammation and Lipid Accumulation through the Polyamine Pathway*. eLife Sciences Publications, Ltd (2025).doi: 10.7554/elife.107841.1.

4. Masuda T, Sankowski R, Staszewski O, Böttcher C, Amann L, Sagar, et al. Spatial and Temporal Heterogeneity of Mouse and Human Microglia at Single-Cell Resolution. *Nature* (2019) 566(7744):388-92. Epub 20190213. doi: 10.1038/s41586-019-0924-x.

5. Doni A, Parente R, Laface I, Magrini E, Cunha C, Colombo FS, et al. Serum Amyloid P Component Is an Essential Element of Resistance against Aspergillus Fumigatus. *Nature Communications* (2021) 12(1):3739. doi: 10.1038/s41467-021-24021-y.

6. Ding Z, Deng Z, Li H. Single-Cell Transcriptome Analysis Reveals the Key Genes Associated with Macrophage Polarization in Liver Cancer. *Hepatol Commun* (2023) 7(11). Epub 20231027. doi: 10.1097/hc9.0000000000000304.

7. Li X, Liu J, Zeng M, Yang K, Zhang S, Liu Y, et al. Gbp2 Promotes M1 Macrophage Polarization by Activating the Notch1 Signaling Pathway in Diabetic Nephropathy. *Front Immunol* (2023) 14:1127612. Epub 20230809. doi: 10.3389/fimmu.2023.1127612.

8. Higgins CB, Adams JA, Ward MH, Greenberg ZJ, Milewska M, Sun J, et al. The Tetraspanin Transmembrane Protein Cd53 Mediates Dyslipidemia and Integrates Inflammatory and Metabolic Signaling in Hepatocytes. *J Biol Chem* (2023) 299(2):102835. Epub 20221227. doi: 10.1016/j.jbc.2022.102835.

9. Gianfranca C, Lara C, Emanuele P, Alessandra C, Enrico T, Lidia B, et al. Polarization Dictates Iron Handling by Inflammatory and Alternatively Activated Macrophages. *Haematologica* (2010) 95(11):1814-22. doi: 10.3324/haematol.2010.023879.

10. Raia S, Conti A, Zanardi A, Ferrini B, Scotti GM, Gilberti E, et al. Ceruloplasmin-Deficient Mice Show Dysregulation of Lipid Metabolism in Liver and Adipose Tissue Reduced by a Protein Replacement. *Int J Mol Sci* (2023) 24(2). Epub 20230106. doi: 10.3390/ijms24021150.

11. Xie L, Yuan Y, Xu S, Lu S, Gu J, Wang Y, et al. Downregulation of Hepatic Ceruloplasmin Ameliorates Nafld Via Sco1-Ampk-Lkb1 Complex. *Cell Rep* (2022) 41(3):111498. doi: 10.1016/j.celrep.2022.111498.

12. Alam S, Liu Q, Liu S, Liu Y, Zhang Y, Yang X, et al. Up-Regulated Cathepsin C Induces Macrophage M1 Polarization through Fak-Triggered P38 Mapk/Nf-Kb Pathway. *Experimental Cell Research* (2019) 382(2):111472. doi: https://doi.org/10.1016/j.yexcr.2019.06.017.

13. Dai J, Liu J, Zhang Q, An Y, Xia B, Wan C, et al. Cathepsin C Is Involved in Macrophage M1 Polarization Via P38/Mapk Pathway in Sudden Cardiac Death. *Cardiovasc Ther* (2021) 2021:6139732. Epub 20211015. doi: 10.1155/2021/6139732.

14. Ruiz-Blázquez P, Pistorio V, Fernández-Fernández M, Moles A. The Multifaceted Role of Cathepsins in Liver Disease. *Journal of Hepatology* (2021) 75(5):1192-202. doi: https://doi.org/10.1016/j.jhep.2021.06.031.

15. Salerno DM, Tront JS, Hoffman B, Liebermann DA. Gadd45a and Gadd45b Modulate Innate Immune Functions of Granulocytes and Macrophages by Differential Regulation of P38 and Jnk Signaling. *J Cell Physiol* (2012) 227(11):3613-20. doi: 10.1002/jcp.24067.

16. Chen M, Menon MC, Wang W, Fu J, Yi Z, Sun Z, et al. Hck Induces Macrophage Activation to Promote Renal Inflammation and Fibrosis Via Suppression of Autophagy. *Nat Commun* (2023) 14(1):4297. Epub 20230718. doi: 10.1038/s41467-023-40086-3.

17. Nguyen VT, Farman N, Palacios-Ramirez R, Sbeih M, Behar-Cohen F, Aractingi S, et al. Cutaneous Wound Healing in Diabetic Mice Is Improved by Topical Mineralocorticoid Receptor Blockade. *J Invest Dermatol* (2020) 140(1):223-34.e7. Epub 20190703. doi: 10.1016/j.jid.2019.04.030.

18. Shen H, Wang Z, Huang A, Zhu D, Sun P, Duan Y. Lipocalin 2 Is a Regulator During Macrophage Polarization Induced by Soluble Worm Antigens. *Front Cell Infect Microbiol* (2021) 11:747135. Epub 20210920. doi: 10.3389/fcimb.2021.747135.

19. Abella V, Scotece M, Conde J, Gómez R, Lois A, Pino J, et al. The Potential of Lipocalin-2/Ngal as Biomarker for Inflammatory and Metabolic Diseases. *Biomarkers* (2015) 20(8):565-71. Epub 20151215. doi: 10.3109/1354750x.2015.1123354.

20. Xu Y, Zhu Y, Jadhav K, Li Y, Sun H, Yin L, et al. Lipocalin-2 Protects against Diet-Induced Nonalcoholic Fatty Liver Disease by Targeting Hepatocytes. *Hepatol Commun* (2019) 3(6):763-75. Epub 20190325. doi: 10.1002/hep4.1341.

21. Zhang ZX, Peng J, Ding WW. Lipocalin-2 and Intestinal Diseases. *World J Gastroenterol* (2024) 30(46):4864-79. doi: 10.3748/wjg.v30.i46.4864.

22. Jiang G, Li J, Niu S, Dong R, Chen Y, Bi W. Ly86 Facilitates Ox-Ldl-Induced Lipid Accumulation in Macrophages by Upregulating Srebp2/Hmgcr Expression. *BMC Cardiovascular Disorders* (2024) 24(1):289. doi: 10.1186/s12872-024-03957-1.

23. Kim KM, Lee KG, Lee S, Hong BK, Yun H, Park YJ, et al. The Acute Phase Reactant Orosomucoid-2 Directly Promotes Rheumatoid Inflammation. *Exp Mol Med* (2024) 56(4):890-903. Epub 20240401. doi: 10.1038/s12276-024-01188-0.

24. Li L, Sun H, Chen J, Ding C, Yang X, Han H, et al. Mitigation of Non-Alcoholic Steatohepatitis Via Recombinant Orosomucoid 2, an Acute Phase Protein Modulating the Erk1/2-Pparγ-Cd36 Pathway. *Cell Reports* (2023) 42(7). doi: 10.1016/j.celrep.2023.112697.

25. Wu X, Wei S, Chen M, Li J, Wei Y, Zhang J, et al. P2ry13 Exacerbates Intestinal Inflammation by Damaging the Intestinal Mucosal Barrier Via Activating Il-6/Stat3 Pathway. *Int J Biol Sci* (2022) 18(13):5056-69. Epub 20220801. doi: 10.7150/ijbs.74304.

26. Duparc T, Gore E, Combes G, Beuzelin D, Da Silva JP, Bouguetoch V, et al. P2y₁₃ Receptor Deficiency Favors Adipose Tissues Lipolysis and Worsens Insulin Resistance and Fatty Liver Disease. *Atherosclerosis* (2024) 395. doi: 10.1016/j.atherosclerosis.2024.118296.

27. de Mezer M, Rogaliński J, Przewoźny S, Chojnicki M, Niepolski L, Sobieska M, et al. Serpina3: Stimulator or Inhibitor of Pathological Changes. *Biomedicines* (2023) 11(1). Epub 20230107. doi: 10.3390/biomedicines11010156.

28. Tran M, Mostofa G, Picard M, Wu J, Wang L, Shin D-J. Serpina3n Deficiency Attenuates Steatosis and Enhances Insulin Signaling in Male Mice. *Journal of Endocrinology* (2023) 256(3). doi: https://doi.org/10.1530/JOE-22-0073.

29. Plevriti A, Lamprou M, Mourkogianni E, Skoulas N, Giannakopoulou M, Sajib MS, et al. The Role of Soluble Cd163 (Scd163) in Human Physiology and Pathophysiology. *Cells* (2024) 13(20). Epub 20241011. doi: 10.3390/cells13201679.

30. Rødgaard-Hansen S, St George A, Kazankov K, Bauman A, George J, Grønbæk H, et al. Effects of Lifestyle Intervention on Soluble Cd163, a Macrophage Activation Marker, in Patients with Non-Alcoholic Fatty Liver Disease. *Scand J Clin Lab Invest* (2017) 77(7):498-504. Epub 20170717. doi: 10.1080/00365513.2017.1346823.

31. Tian HY, Liang Q, Shi Z, Zhao H. Exosomal Cxcl14 Contributes to M2 Macrophage Polarization through Nf-Kb Signaling in Prostate Cancer. *Oxid Med Cell Longev* (2022) 2022:7616696. Epub 20220527. doi: 10.1155/2022/7616696.

32. AHMAD R, KOCHUMON SP, BAHMAN F, AREFANIAN H, SHENOUDA S, JACOB TK, et al. 193-Or: Il-6 Deficiency Mitigates Metabolic Dysregulation in Mice Via Cxcl14-Driven Inflammation Reduction in Adipose and Liver Tissues. *Diabetes* (2025) 74(Supplement_1). doi: 10.2337/db25-193-OR.

33. Wang Z, Fu J, Zhu S, Tang H, Shi K, Yang J, et al. Identification of M2 Macrophage-Related Genes for Establishing a Prognostic Model in Pancreatic Cancer: Fcgr3a as Key Gene. *Oncol Res* (2024) 32(12):1851-66. Epub 20241113. doi: 10.32604/or.2024.055286.

34. Zhang M, Nakamura K, Kageyama S, Lawal AO, Gong KW, Bhetraratana M, et al. Myeloid Ho-1 Modulates Macrophage Polarization and Protects against Ischemia-Reperfusion Injury. *JCI Insight* (2018) 3(19). Epub 20181004. doi: 10.1172/jci.insight.120596.

35. Dunn LL, Midwinter RG, Ni J, Hamid HA, Parish CR, Stocker R. New Insights into Intracellular Locations and Functions of Heme Oxygenase-1. *Antioxid Redox Signal* (2014) 20(11):1723-42. Epub 20140228. doi: 10.1089/ars.2013.5675.

36. Chen H-H, Keyhanian K, Zhou X, Vilmundarson RO, Almontashiri NAM, Cruz SA, et al. Irf2bp2 Reduces Macrophage Inflammation and Susceptibility to Atherosclerosis. *Circulation Research* (2015) 117(8):671-83. doi: doi:10.1161/CIRCRESAHA.114.305777.

37. Fang J, Ji YX, Zhang P, Cheng L, Chen Y, Chen J, et al. Hepatic Irf2bp2 Mitigates Nonalcoholic Fatty Liver Disease by Directly Repressing the Transcription of Atf3. *Hepatology* (2020) 71(5):1592-608. Epub 20200130. doi: 10.1002/hep.30950.

38. Liu YY, Rajkumar K, Murphy LJ. Hepatic Regeneration in Insulin-Like Growth Factor Binding Protein-1 Transgenic Mice. *J Hepatol* (1999) 30(4):674-80. doi: 10.1016/s0168-8278(99)80199-8.

39. Lewitt MS, Boyd GW. Insulin-Like Growth Factor-Binding Protein-1 (Igfbp-1) as a Biomarker of Cardiovascular Disease. *Biomolecules* (2024) 14(11). Epub 20241120. doi: 10.3390/biom14111475.

40. Meyer NMT, Kabisch S, Dambeck U, Honsek C, Kemper M, Gerbracht C, et al. Igf-1 and Igfbp-1 as Possible Predictors of Response to Lifestyle Intervention-Results from Randomized Controlled Trials. *Int J Mol Sci* (2024) 25(12). Epub 20240610. doi: 10.3390/ijms25126400.

41. Spadaro O, Camell CD, Bosurgi L, Nguyen KY, Youm Y-H, Rothlin CV, et al. Igf1 Shapes Macrophage Activation in Response to Immunometabolic Challenge. *Cell Reports* (2017) 19(2):225-34. doi: https://doi.org/10.1016/j.celrep.2017.03.046.

42. Kumar Talari N, Mattam U, Kaminska D, Sotomayor-Rodriguez I, Rahman A, Péterfy M, et al. Hepatokine Itih3 Protects against Hepatic Steatosis by Downregulating Mitochondrial Bioenergetics and De Novo Lipogenesis. *Physiology* (2024) 39(S1):352. doi: 10.1152/physiol.2024.39.S1.352.

43. Talari NK, Mattam U, Kaminska D, Sotomayor-Rodriguez I, Rahman AP, Péterfy M, et al. Hepatokine Itih3 Protects against Hepatic Steatosis by Downregulating Mitochondrial Bioenergetics and <Em>De Novo</Em> Lipogenesis. *iScience* (2024) 27(5). doi: 10.1016/j.isci.2024.109709.

44. Fujita Y, Ezura Y, Emi M, Sato K, Takada D, Iino Y, et al. Hypercholesterolemia Associated with Splice-Junction Variation of Inter-A-Trypsin Inhibitor Heavy Chain 4 (Itih4) Gene. *Journal of Human Genetics* (2004) 49(1):24-8. doi: 10.1007/s10038-003-0101-8.

45. Zhao X, Guo Y, Li L, Li Y. Longitudinal Change of Serum Inter-Alpha-Trypsin Inhibitor Heavy Chain H4, and Its Correlation with Inflammation, Multiorgan Injury, and Death Risk in Sepsis. *J Clin Lab Anal* (2023) 37(3):e24834. Epub 20230201. doi: 10.1002/jcla.24834.

46. Hamada M, Tsunakawa Y, Jeon H, Yadav MK, Takahashi S. Role of Mafb in Macrophages. *Exp Anim* (2020) 69(1):1-10. Epub 20191001. doi: 10.1538/expanim.19-0076.

47. Yu H, Jiang HL, Xu D, Jin JZ, Zhao ZM, Ma YD, et al. Transcription Factor Mafb Promotes Hepatocellular Carcinoma Cell Proliferation through up-Regulation of Cyclin D1. *Cell Physiol Biochem* (2016) 39(2):700-8. Epub 20160725. doi: 10.1159/000445661.

48. Yue L, Xu X, Dai S, Xu F, Zhao W, Gu J, et al. Orosomucoid 1 Promotes Colorectal Cancer Progression and Liver Metastasis by Affecting Pi3k/Akt Pathway and Inducing Macrophage M2 Polarization. *Scientific Reports* (2023) 13(1):14092. doi: 10.1038/s41598-023-40404-1.

49. Lee YS, Choi JW, Hwang I, Lee JW, Lee JH, Kim AY, et al. Adipocytokine Orosomucoid Integrates Inflammatory and Metabolic Signals to Preserve Energy Homeostasis by Resolving Immoderate Inflammation*. *Journal of Biological Chemistry* (2010) 285(29):22174-85. doi: https://doi.org/10.1074/jbc.M109.085464.

50. Jiang J, Chen H, Zhao C, Li T, Zhang C, Ma L, et al. Prtn3 Promotes Il33/Treg-Mediated Tumor Immunosuppression by Enhancing the M2 Polarization of Tumor-Associated Macrophages in Lung Adenocarcinoma. *Cancer Letters* (2025) 616:217584. doi: https://doi.org/10.1016/j.canlet.2025.217584.

51. Toonen EJ, Mirea AM, Tack CJ, Stienstra R, Ballak DB, van Diepen JA, et al. Activation of Proteinase 3 Contributes to Non-Alcoholic Fatty Liver Disease (Nafld) and Insulin Resistance. *Mol Med* (2016) 22:202-14. Epub 20160524. doi: 10.2119/molmed.2016.00033.

52. Arbiser JL. A Tale of Two Macrophages: To Shb or Not to Shb. *Acta Physiol (Oxf)* (2015) 214(2):152-3. Epub 20150420. doi: 10.1111/apha.12504.

53. Sheng L, Liu Y, Jiang L, Chen Z, Zhou Y, Cho KW, et al. Hepatic Sh2b1 and Sh2b2 Regulate Liver Lipid Metabolism and Vldl Secretion in Mice. *PLoS One* (2013) 8(12):e83269. Epub 20131217. doi: 10.1371/journal.pone.0083269.

54. Panda SK, Kim DH, Desai P, Rodrigues PF, Sudan R, Gilfillan S, et al. Slc7a8 Is a Key Amino Acids Supplier for the Metabolic Programs That Sustain Homeostasis and Activation of Type 2 Innate Lymphoid Cells. *Proc Natl Acad Sci U S A* (2022) 119(46):e2215528119. Epub 20221107. doi: 10.1073/pnas.2215528119.

55. Zhang C, Yang X, Xue Y, Li H, Zeng C, Chen M. The Role of Solute Carrier Family Transporters in Hepatic Steatosis and Hepatic Fibrosis. *J Clin Transl Hepatol* (2025) 13(3):233-52. Epub 20250122. doi: 10.14218/jcth.2024.00348.

56. Zhu L, Wang Z, Han W, Xu A. Comprehensive Analysis of the Biological Function and Immune Infiltration of Slc38a2 in Gastric Cancer. *BMC Gastroenterol* (2023) 23(1):74. Epub 20230314. doi: 10.1186/s12876-023-02689-4.

57. Gauthier-Coles G, Bröer A, McLeod MD, George AJ, Hannan RD, Bröer S. Identification and Characterization of a Novel Snat2 (Slc38a2) Inhibitor Reveals Synergy with Glucose Transport Inhibition in Cancer Cells. *Frontiers in Pharmacology* (2022) Volume 13 - 2022. doi: 10.3389/fphar.2022.963066.

58. Huang Z, Li Y, Liu Q, Chen X, Lin W, Wu W, et al. Spp1-Mediated M2 Macrophage Polarization Shapes the Tumor Microenvironment and Enhances Prognosis and Immunotherapy Guidance in Nasopharyngeal Carcinoma. *International Immunopharmacology* (2025) 147:113944. doi: https://doi.org/10.1016/j.intimp.2024.113944.

59. Han H, Ge X, Komakula SSB, Desert R, Das S, Song Z, et al. Macrophage-Derived Osteopontin (<Em>Spp1</Em>) Protects from Nonalcoholic Steatohepatitis. *Gastroenterology* (2023) 165(1):201-17. doi: 10.1053/j.gastro.2023.03.228.

60. Liu J, Zhang W, Chen L, Wang X, Mao X, Wu Z, et al. Vsig4 Promotes Tumour-Associated Macrophage M2 Polarization and Immune Escape in Colorectal Cancer Via Fatty Acid Oxidation Pathway. *Clinical and Translational Medicine* (2025) 15(5):e70340. doi: https://doi.org/10.1002/ctm2.70340.

61. Liang X, Li P, Jiang J, Xin J, Luo J, Li J, et al. Transcriptomics Unveils Immune Metabolic Disruption and a Novel Biomarker of Mortality in Patients with Hbv-Related Acute-on-Chronic Liver Failure. *JHEP Reports* (2023) 5(9):100848. doi: https://doi.org/10.1016/j.jhepr.2023.100848.

62. Bahman F, Choudhry K, Al-Rashed F, Al-Mulla F, Sindhu S, Ahmad R. Aryl Hydrocarbon Receptor: Current Perspectives on Key Signaling Partners and Immunoregulatory Role in Inflammatory Diseases. *Frontiers in Immunology* (2024) Volume 15 - 2024. doi: 10.3389/fimmu.2024.1421346.

63. Tanos R, Murray IA, Smith PB, Patterson A, Perdew GH. Role of the Ah Receptor in Homeostatic Control of Fatty Acid Synthesis in the Liver. *Toxicol Sci* (2012) 129(2):372-9. Epub 20120613. doi: 10.1093/toxsci/kfs204.

64. Chistiakov DA, Killingsworth MC, Myasoedova VA, Orekhov AN, Bobryshev YV. Cd68/Macrosialin: Not Just a Histochemical Marker. *Laboratory Investigation* (2017) 97(1):4-13. doi: 10.1038/labinvest.2016.116.

65. Liu R, Scimeca M, Sun Q, Melino G, Mauriello A, Shao C, et al. Harnessing Metabolism of Hepatic Macrophages to Aid Liver Regeneration. *Cell Death & Disease* (2023) 14(8):574. doi: 10.1038/s41419-023-06066-7.

66. McCormick SM, Gowda N, Fang JX, Heller NM. Suppressor of Cytokine Signaling (Socs)1 Regulates Interleukin-4 (Il-4)-Activated Insulin Receptor Substrate (Irs)-2 Tyrosine Phosphorylation in Monocytes and Macrophages Via the Proteasome *. *Journal of Biological Chemistry* (2016) 291(39):20574-87. doi: 10.1074/jbc.M116.746164.

67. Krause C, Geißler C, Tackenberg H, El Gammal AT, Wolter S, Spranger J, et al. Multi-Layered Epigenetic Regulation of Irs2 Expression in the Liver of Obese Individuals with Type 2 Diabetes. *Diabetologia* (2020) 63(10):2182-93. doi: 10.1007/s00125-020-05212-6.

68. Gaiser AK, Bauer S, Ruez S, Holzmann K, Fändrich M, Syrovets T, et al. Serum Amyloid A1 Induces Classically Activated Macrophages: A Role for Enhanced Fibril Formation. *Front Immunol* (2021) 12:691155. Epub 20210630. doi: 10.3389/fimmu.2021.691155.

69. Anthony D, McQualter JL, Bishara M, Lim EX, Yatmaz S, Seow HJ, et al. Saa Drives Proinflammatory Heterotypic Macrophage Differentiation in the Lung Via Csf-1r-Dependent Signaling. *The FASEB Journal* (2014) 28(9):3867-77. doi: https://doi.org/10.1096/fj.14-250332.

70. Mohanty T, Miličević K, Göthert H, Tillmann A, Padra M, Papareddy P, et al. Balancing Inflammation: The Specific Roles of Serum Amyloid a Proteins in Sterile and Infectious Diseases. *Frontiers in Immunology* (2025) Volume 16 - 2025. doi: 10.3389/fimmu.2025.1544085.

71. Ji A, Meredith LW, Shridas P. Serum Amyloid A: A Double-Edged Sword in Health and Disease. *International Journal of Molecular Sciences* (2025) 26(10):4528.

72. de Beer MC, Yuan T, Kindy MS, Asztalos BF, Roheim PS, de Beer FC. Characterization of Constitutive Human Serum Amyloid a Protein (Saa4) as an Apolipoprotein. *Journal of Lipid Research* (1995) 36(3):526-34. doi: https://doi.org/10.1016/S0022-2275(20)39886-2.

73. Liu A-r, Liu Y-n, Shen S-x, Yan L-r, Lv Z, Ding H-x, et al. Comprehensive Analysis and Validation of Solute Carrier Family 25 (Slc25) and Its Correlation with Immune Infiltration in Pan-Cancer. *BioMed Research International* (2022) 2022(1):4009354. doi: https://doi.org/10.1155/2022/4009354.

74. Tavares CDJ, Aigner S, Sharabi K, Sathe S, Mutlu B, Yeo GW, et al. Transcriptome-Wide Analysis of Pgc-1α-Binding Rnas Identifies Genes Linked to Glucagon Metabolic Action. *Proc Natl Acad Sci U S A* (2020) 117(36):22204-13. Epub 20200826. doi: 10.1073/pnas.2000643117.

75. Lang L, Zheng D, Jiang Q, Meng T, Ma X, Yang Y. Uncoupling Protein 2 Modulates Polarization and Metabolism of Human Primary Macrophages Via Glycolysis and the Nf-Kb Pathway. *Exp Ther Med* (2023) 26(6):583. doi: 10.3892/etm.2023.12282.

Supplementary *Figure 5*. ***Heatmap for DEG across the groups that are involving in inflammation and metabolism***

Supplementary *Figure 5*

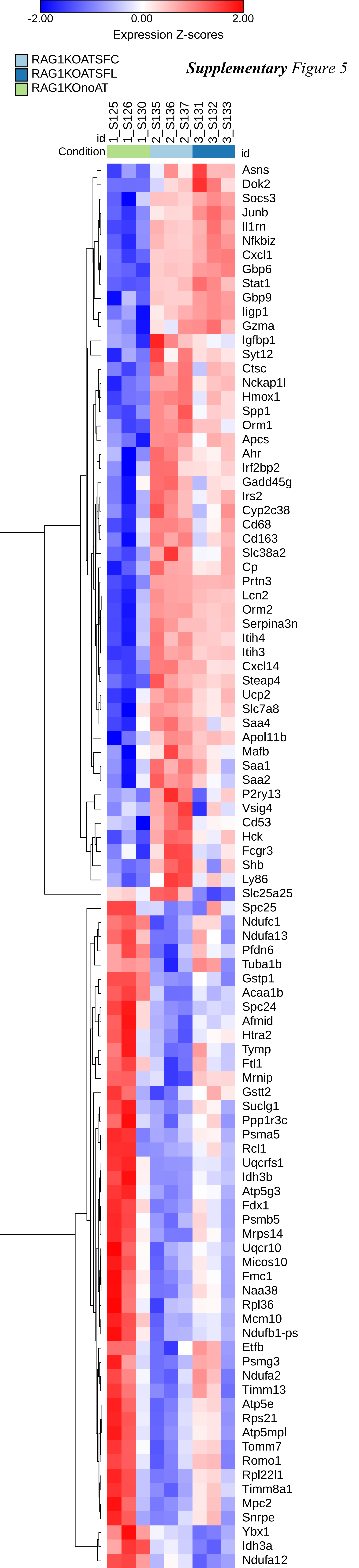

Supplementary** Figure 6**. Gut microbiota analysis-Diversity

Alpha-Diversity: Faith_PD, Observed_Species, Shannon, and Evenness

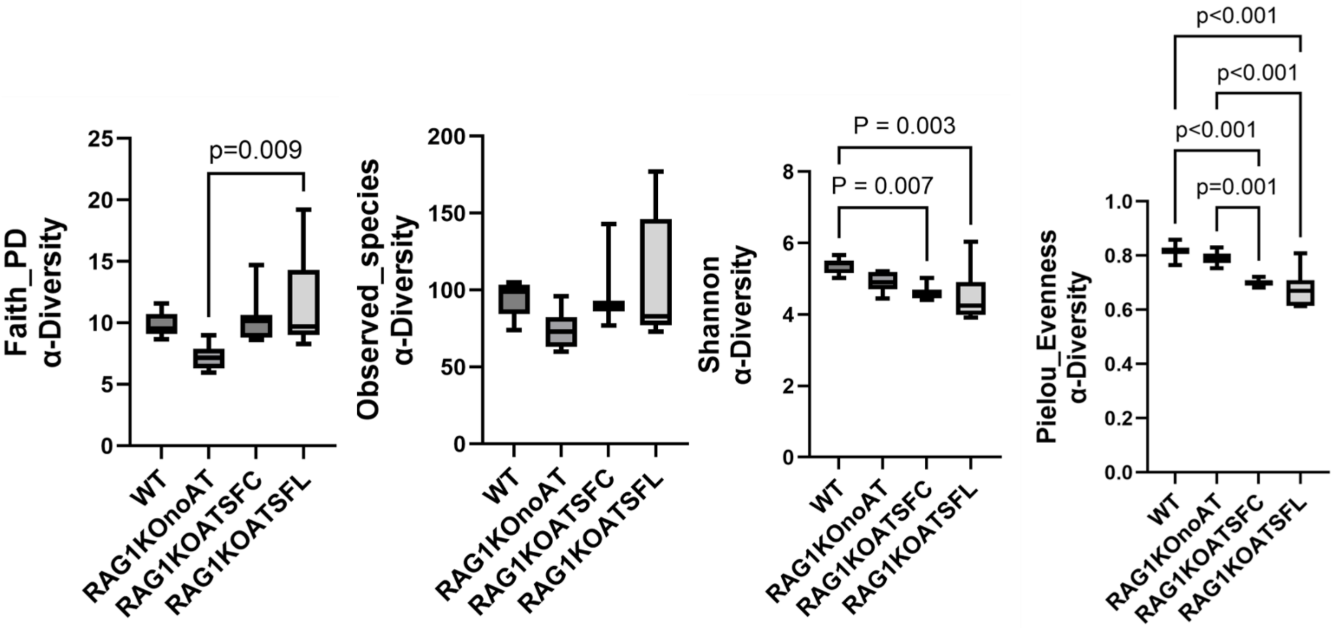

Supplementary *Figure 7*. ***Gut microbiota analysis-Taxa at genus level***

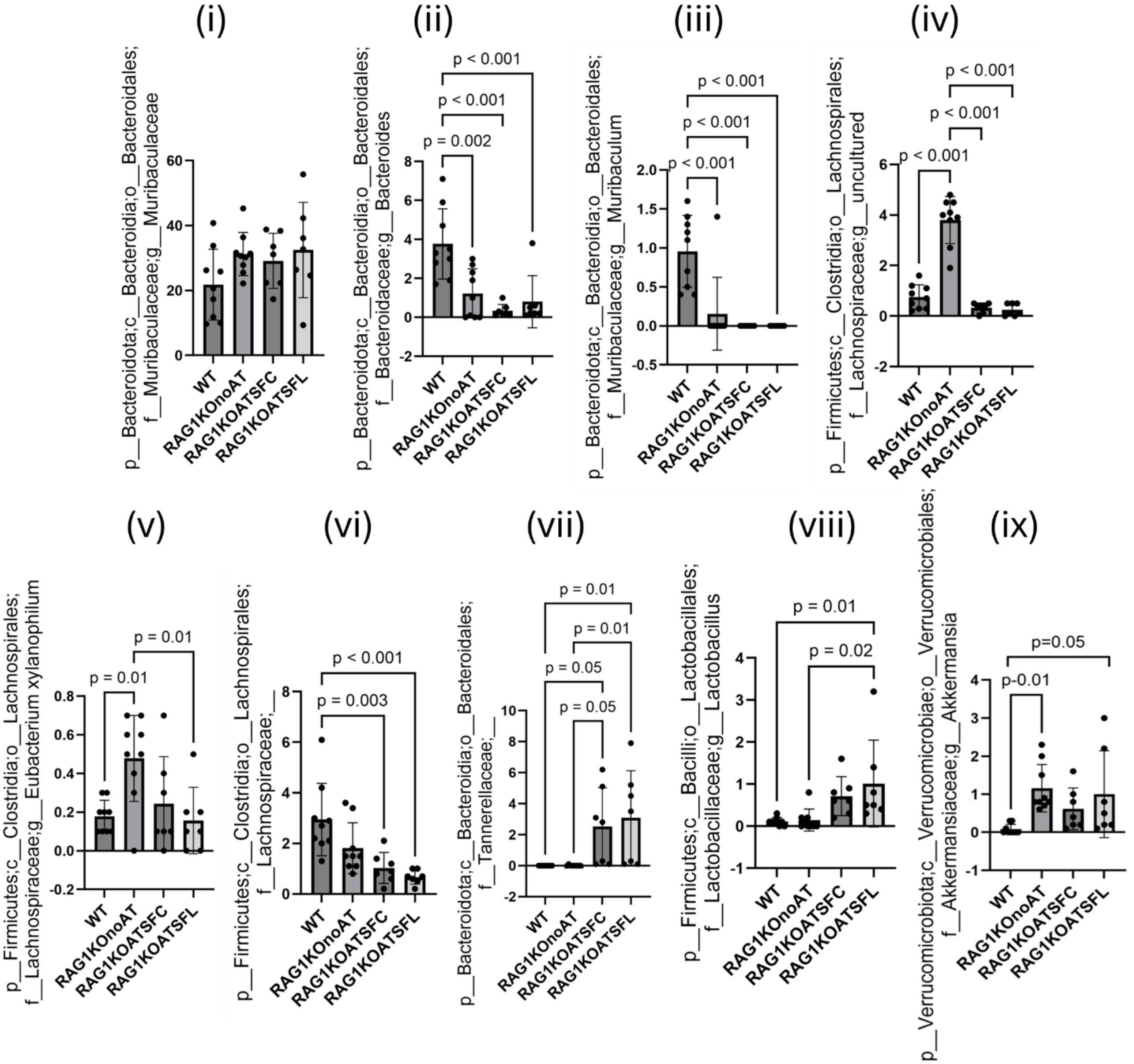

Supplementary** Table 5**. The DEGs across the groups that are demonstrated in Figures (see an attached excel table)

Supplementary** Table 6**. The full DEG list with log2FC and significant adj p values in the comparison of RAG1KOATSFC vs. RAG1KOnoAT (see an attached excel table)

Supplementary** Table 7**. The full DEG list with log2FC and significant adj p values in the comparison of RAG1KOATSFL vs. RAG1KOATSFC (see an attached excel table)
